# IĸB Protein BCL3 as a Controller of Osteogenesis and Bone Health

**DOI:** 10.1002/art.42639

**Published:** 2023-10-01

**Authors:** Hussain Jaffery, Carmen Huesa, Sabarinadh Chilaka, John Cole, James Doonan, Moeed Akbar, Lynette Dunning, Kathleen Elizabeth Tanner, Rob J. van ‘t Hof, Iain B. McInnes, Ruaidhrí J. Carmody, Carl S. Goodyear

**Affiliations:** ^1^ School of Infection & Immunity University of Glasgow Glasgow UK; ^2^ School of Infection & Immunity, University of Glasgow, Glasgow and Institute of Biomedical & Environmental Health, University of the West of Scotland Paisley UK; ^3^ Institute of Biomedical & Environmental Health University of the West of Scotland Paisley UK; ^4^ James Watt School of Engineering University of Glasgow Glasgow UK; ^5^ Present address: School of Engineering and Materials Science and Institute of Bioengineering Queen Mary University of London London UK; ^6^ Institute of Ageing and Chronic Disease University of Liverpool Liverpool UK

## Abstract

**Objective:**

IĸB protein B cell lymphoma 3‐encoded protein (BCL3) is a regulator of the NF‐κB family of transcription factors. NF‐κB signaling fundamentally influences the fate of bone‐forming osteoblasts and bone‐resorbing osteoclasts, but the role of BCL3 in bone biology has not been investigated. The objective of this study was to evaluate BCL3 in skeletal development, maintenance, and osteoarthritic pathology.

**Methods:**

To assess the contribution of BCL3 to skeletal homeostasis, neonatal mice (n = 6–14) lacking BCL3 (*Bcl3* ^*−/−*^) and wild‐type (WT) controls were characterized for bone phenotype and density. To reveal the contribution to bone phenotype by the osteoblast compartment in *Bcl3* ^*−/−*^ mice, transcriptomic analysis of early osteogenic differentiation and cellular function (n = 3–7) were assessed. Osteoclast differentiation and function in *Bcl3* ^*−/−*^ mice (n = 3–5) was assessed. Adult 20‐week *Bcl3* ^*−/−*^ and WT mice bone phenotype, strength, and turnover were assessed. A destabilization of the medial meniscus model of osteoarthritic osteophytogenesis was used to understand adult bone formation in *Bcl3* ^*−/−*^ mice (n = 11–13).

**Results:**

Evaluation of *Bcl3* ^*−/−*^ mice revealed congenitally increased bone density, long bone dwarfism, increased bone biomechanical strength, and altered bone turnover. Molecular and cellular characterization of mesenchymal precursors showed that *Bcl3* ^*−/−*^ cells displayed an accelerated osteogenic transcriptional profile that led to enhanced differentiation into osteoblasts with increased functional activity, which could be reversed with a mimetic peptide. In a model of osteoarthritis‐induced osteophytogenesis, *Bcl3* ^*−/−*^ mice exhibited decreased pathological osteophyte formation (*P* < 0.05).

**Conclusion:**

Cumulatively, these findings demonstrate that BCL3 controls developmental mineralization to enable appropriate bone formation, whereas in a pathological setting, it contributes to skeletal pathology.

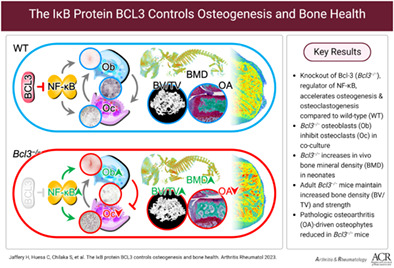

## INTRODUCTION

Homeostatic bone growth and subsequent skeletal maintenance is a tightly controlled process that relies on the coordination of osteoblasts (depositing mineralized matrix) and osteoclasts (resorbing matrix) (1). The differentiation, interaction, and ensuing function of these cells are regulated by both extracellular factors (e.g., metabolites, bone morphogenetic proteins [BMPs], fibroblast growth factors, Wnt family proteins, macrophage colony‐stimulating factor [M‐CSF], and RANKL) (2, 3) and intracellular factors, including NF‐κB (4). Perturbation of this closely regulated system can contribute to decreased bone quality and pathology observed in diseases such as osteoarthritis, rheumatoid arthritis, and osteoporosis.

The NF‐κB family of transcription factors is critical in determining the differentiation and activities of cells affecting skeletal development and maintenance. Combined, this family functions as a master regulator of gene transcription. Members are characterized by the presence of the Rel homology domain, which mediates dimerization and DNA binding (5). NF‐κB activation involves a cascade of events, including receptor ligation, activation of IκB kinases (IKKs) and phosphorylation, and degradation of IκBs, which ultimately triggers the nuclear translocation of NF‐κB dimers (5). The generation of homodimers or heterodimers is based on the interaction of five subunits (p65/RelA, RelB, c‐Rel, p50/NF‐κB1, and p52/NF‐κB2) that can bind to specific DNA sequences (κB sites) in the target promoters of hundreds of genes (6). Notably, homodimers of the p50 and p52 NF‐κB subunits, which lack transcription activation domains, interact with the atypical IκB protein B cell lymphoma 3‐encoded protein (BCL3) (5). BCL3, initially identified as a proto‐oncogene, is a predominantly nuclear protein containing seven ankyrin repeat domains (5). Upon tumor necrosis factor (TNF) receptor or toll‐like receptor‐driven NF‐κB activation, BCL3 stabilizes p50 homodimers at NF‐κB binding sites (κB sites), preventing p50 degradation and competitively inhibiting transactivating p65 or c‐Rel containing NF‐κB dimers from occupying the κB sites (5). Moreover, it has been speculated that the interaction of BCL3 with homodimers of p50 and p52 leads to recruitment of additional transcriptional corepressors or coactivators (5). The physiological role of Bcl3 is well described as a regulator of adaptive immunity through control of T and B cell maturation and formation of lymph node germinal centers (7).

Prior studies have investigated the role of NF‐κB in osteoblast and osteoclast differentiation and function. In osteoblasts, NF‐κB activation has been reported to both induce and inhibit osteogenesis. For instance, TNF‐driven NF‐κB activation can reduce bone formation by inhibiting Runt‐related transcription factor 2 (RUNX2), an osteoblast lineage–specific transcription factor, which subsequently disrupts BMP signaling (8). Activity of the IKK complex has been implicated in inhibiting osteoblast activity (9). Furthermore, disruption of NF‐κB–inducing kinase (NIK) results in increased p100 levels, leading to increased bone formation and osteoblast numbers, whereas disruption of RelB increases bone formation (10, 11). In juxtaposition, activation of NF‐κB can enhance early osteogenesis, and p65/RelA is essential for osteoblast activity and survival (12, 13). Finally, low‐dose TNF can increase osteogenesis via NF‐κB activation (14). Taken together, this illustrates that the role of NF‐κB activity in regulating osteoblast differentiation is complex and context specific.

In osteoclast differentiation, the binding of RANKL to RANK leads to NF‐κB activation (4). Notably, the RANK gene *Tnfrsf11a* possesses κB sites at the proximal promoter region, which facilitates a feed‐forward RANKL response (15). Furthermore, RelB/p65 dimers strongly induce the central osteoclastogenic transcription factor NFATc1, thereby increasing osteoclast differentiation, survival, and function (4). Mice lacking the NF‐κB p50 and p52 subunits exhibit severe dwarfism, a thickened hypertrophic chondrocyte layer, severe osteopetrosis, and an absence of osteoclasts (16).

Although it is accepted that NF‐κB plays an essential role in bone biology, the specific contribution of BCL3 in shaping bone is ill defined. Herein, we show that BCL3‐deficient (*Bcl3* ^*−/−*^) neonatal mice have congenitally increased bone mineral density and truncated long bones compared to wild‐type (WT) counterparts. The observed increased bone matrix in *Bcl3* ^*−/−*^ mice is explained by an acceleration of the osteogenic transcriptional program. This increase in bone density is maintained into maturation (20‐week‐old mice); however, at this stage, although there is increased bone density and associated biomechanical strength, new bone formation is inhibited. Finally, in a model of pathological osteophytogenesis, we demonstrate that *Bcl3* ^*−/−*^ mice have reduced ectopic osteophyte formation. Collectively, our data illustrate that BCL3 is an intrinsic regulator of osteoblast and osteoclast differentiation and thus a modulator of skeletal health.

## MATERIALS AND METHODS

Detailed information for the following materials and methods for quantitative polymerase chain reaction (PCR), osteoclast differentiation, and functional assessment are described in the Supplementary Methods, available on the *Arthritis & Rheumatology* website at http://onlinelibrary.wiley.com/doi/10.1002/art.42639.

### Animals

*Bcl3* ^*−/−*^ and WT littermates were bred under standard conditions (17, 18). All animal studies received ethical approval from the University of Glasgow and were performed under a UK Home Office license.

### Micro–computed tomography analysis of neonates

Postnatal day 0, mice were scanned via micro–computed tomography (micro‐CT) (4.5‐μm resolution). Reconstructions were manually analyzed using DataViewer (version 1.5.2.4) Visualizations were rendered in CTvox (version 3.1.1).

### Calvarial osteoblast isolation and differentiation

Calvaria were dissected from neonatal mice (postnatal days 3–5) and digested in collagenase II solution (Gibco). Cells were expanded for three days, subsequently seeded at 1.25 × 10^4^ cells/well, and cultured for an additional three days. Osteogenic Medium (supplemented with 50 μg/ml of ascorbic acid and 2 m*M* β‐glycerophosphate) was added to initiate osteogenesis (day 0). In certain cultures, 30 μ*M* BCL3 mimetic peptide (BDP2) or mutated peptide (mBDP2) was added.

### RNA sequencing library preparation and sequencing

Cells were lysed using 700 μl of QIAzol Lysis Reagent (QIAGEN). RNA purification was performed as per standard manufacturer instructions using the miRNeasy Mini Kit (QIAGEN). Complementary DNA synthesis was conducted using the SMART‐seq v4 Ultra Low Input RNA Kit for Sequencing (Clontech). Library preparation was conducted using the Low Input Library Prep Kit HT (Clontech) as per instructions. Sequencing was conducted using the Illumina HiSeq 4000 System with a read depth of ~36 million/sample of 75‐bp paired‐end reads.

### Bioinformatic analysis

Differential expression between the four groups was calculated using DESeq2. Final expression values were represented as fragments per kilobase of exon per million reads. A statistical cutoff of an adjusted *P* value less than 0.05 was considered significant. A downstream analysis used the PANTHER gene ontology (GO) enrichment analysis tool (19). A transcription factor binding site analysis was conducted of the region 350 bp upstream and 50 bp downstream of the gene transcription start site using HOMER (20). A protein–protein functional association network clustering analysis involved Markov clustering algorithm with a 1.2 inflation parameter (21). RNA sequencing data are deposited in NCBI's Gene Expression Omnibus (GEO) and are accessible through GEO Series accession number GSE125153 (https://www.ncbi.nlm.nih.gov/geo/query/acc.cgi?acc=GSE125153).

### Histological assessment of osteoblast cultures

Alkaline phosphatase assay and Alizarin Red staining was conducted as previously described (22). Sircol staining was performed on fixed cells using Sircol Sirius Red (Biocolor) soluble dye solution. Image analysis was conducted via Fiji/ImageJ (version 2.0) packages and involved thresholding of dark pixels (23).

### Micro‐CT analysis of adult long bones

Micro‐CT images of whole fixed deskinned adult legs were acquired on the SkyScan 1272 micro‐CT scanner (Bruker). Images were processed by a back‐projection method using Nrecon (version 1.6.10.4). Specialized macros for the trabecular and cortical regions in the CT Analyzer (version 1.16.4.1) software enabled quantitative analysis on the various volumes.

### Biomechanical analyses

Tibiae were assessed in three‐point flexure (bend) tests using a 50N load cell affixed to a calibrated three‐point apparatus (Zwick Roell) with a 10‐mm/min force advancement, and data were recorded until fracture. Femurs were placed in a 2‐kN load cell (Zwick Roell) with a displacement rate of 0.1 mm/s, and data were recorded until fracture occurred.

### Dynamic histomorphometry and histology

Mice were intraperitoneally injected with 15 mg/kg of calcein dye (Sigma‐Aldrich) solution in Dulbecco's phosphate buffered saline on days −5 and −2 prior to harvest. Femurs were fixed, embedded in methyl methacrylate resin, and cut into 5‐μm sections. For dynamic histomorphometry, slides were counterstained with 1% Calcein Blue (Sigma‐Aldrich), and fluorescent intra‐label thickness was quantified. For histological assessment of the growth plate, slides were dual stained with Safranin O and Fast Green.

### Bone turnover enzyme‐linked immunosorbent assays

Enzyme immunoassays were used to detect serum levels of procollagen type 1 amino‐terminal peptide (P1NP) and carboxyl‐terminal crosslinks peptide of type 1 collagen (CTX1) (Immunodiagnostic Systems) and osteoprotegerin (OPG) and RANKL (R&D Systems) according to the manufacturer's instructions.

### Experimental osteoarthritis model

Destabilization of the medial meniscus (DMM) (on a 10‐week‐old male) and subsequent micro‐CT analysis were performed as previously described (24).

### Statistical approach

For statistical analysis, GraphPad Prism (versions 7–9) and R packages were used. *P* values and adjusted *P* values less than 0.05 were considered significant.

## RESULTS

### BCL3 alters skeletal development

**Figure 1:**
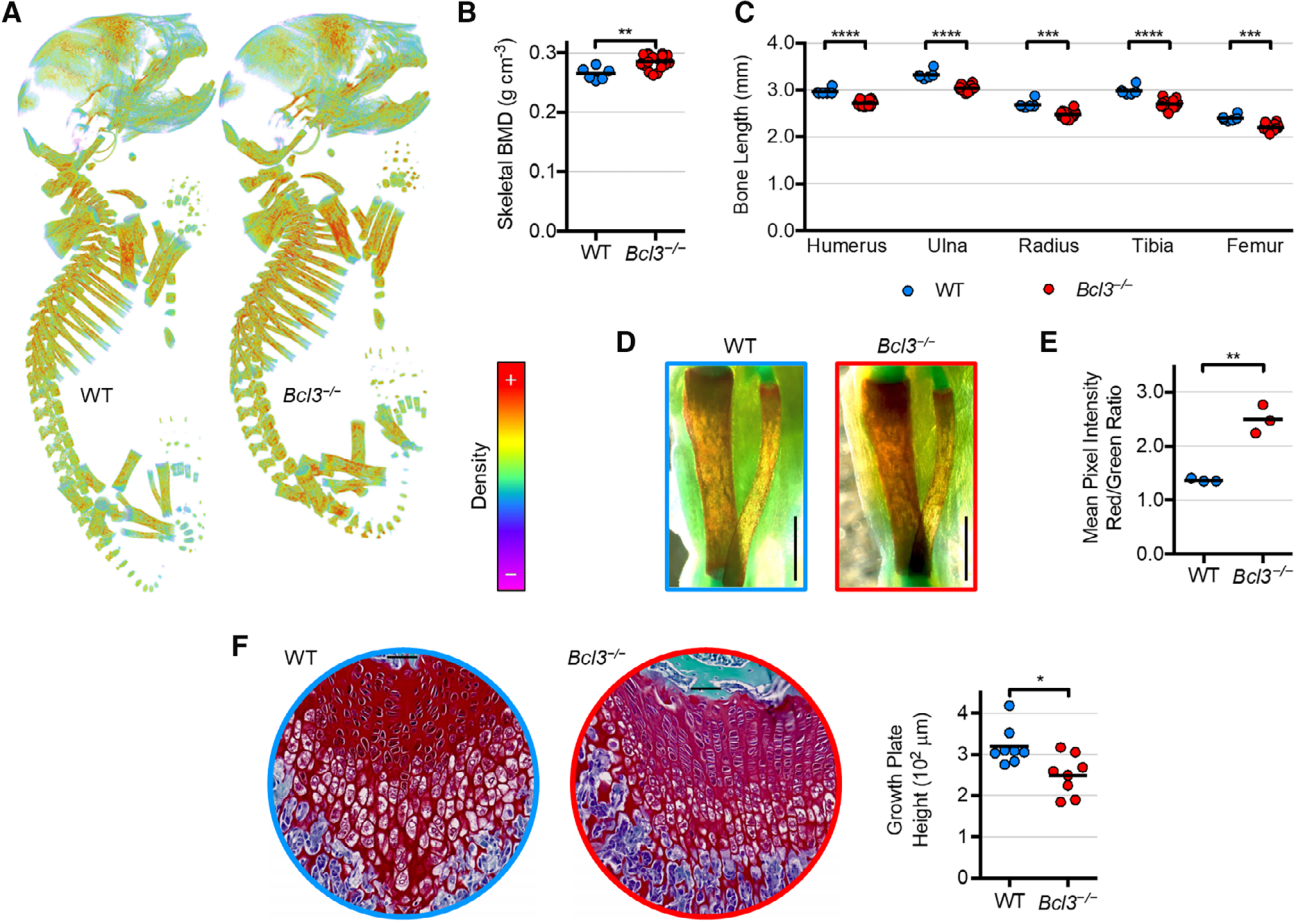
Whole skeletal and long bone assessment of wild‐type (WT) and *Bcl3* ^*−/−*^ neonatal (P0) mice using micro–computed tomography (micro‐CT) and whole‐mount stains. **A**, Representative images of neonatal three‐dimensional projection models of bone mineral density (BMD). Coloration depicts high‐intensity voxels as red and low‐intensity voxels as violet. Images are not to scale. **B**, Whole skeletal BMD. n = 6 (WT) and 14 (*Bcl3* ^*−/−*^). **C**, Longitudinal length measurements comparing major WT and *Bcl3* ^*−/−*^ appendicular long bones of neonates using micro‐CT. n = 6–14. **D**, Tibiae and fibulae of the right legs of individual whole‐mount Alizarin Red S and Alcian Blue–stained mice viewed medially in brightfield. Scale bars represent 1 mm. Student's *t*‐test was used. **E**, Red‐to‐green mean pixel intensity ratio of tibial diaphysis staining, with red denoting Ca^2+^ mineral and green denoting background. **F**, Histological dual stain (Safranin O and Fast Green) of femurs from 4‐week‐old WT and *Bcl3* ^*−/−*^ male mice. Representative images are shown. Image diameter = 500 μm, and inset horizontal bar = 50 μm. n = 7–8. Student's *t*‐test (**B** and **E**) or the Mann‐Whitney test (**C** and **F**) was used. * *P* < 0.05; ** *P* < 0.01; *** *P* < 0.001; **** *P* < 0.0001.

To investigate whether congenital skeletal abnormalities are associated with loss of BCL3, neonatal (postnatal day 0) WT and *Bcl3* ^*−/−*^ mice were examined by x‐ray micro‐CT (Figure 1A). Gross skeletal abnormalities were absent in *Bcl3* ^*−/−*^ pups; however, analysis of the complete skeleton revealed that *Bcl3* ^*−/−*^ neonatal mice had an increase in overall bone mineral density relative to WT mice (Figure 1B). This was also associated with a significant reduction in the length of mineralized long bones (including the humerus, ulna, radius, tibia, and femur) in *Bcl3* ^*−/−*^ mice compared to WT controls (Figure 1C).

Further interrogation of glycan‐rich cartilage (Alcian Blue staining) and calcium mineral (Alizarin Red staining) in whole‐mount skeletons uncovered altered skeletal composition in *Bcl3* ^*−/−*^ neonatal mice. Notably, *Bcl3* ^*−/−*^ neonates had demonstrably increased Alizarin Red staining in their long bones (e.g., tibia) compared to WT controls, indicative of increased bone mineral density and osteoblast hyperactivity (Figure 1D). This was further supported by specific quantitative analysis of Alizarin Red staining in the tibial diaphysis (Figure 1E). A histological analysis (Safranin O and Fast Green staining) revealed a decrease in the cartilaginous growth plate height of 4‐week‐old *Bcl3* ^*−/−*^ mice compared to WT controls, indicative of perturbation of the osteochondral lineage (Figure 1F). Combined, these data suggest that loss of BCL3 results in overactive mineralization coupled with reduced overall long bone growth.

### *Bcl3* ^*−/−*^ osteoblast transcriptome shows an accelerated early osteogenesis signature

**Figure 2:**
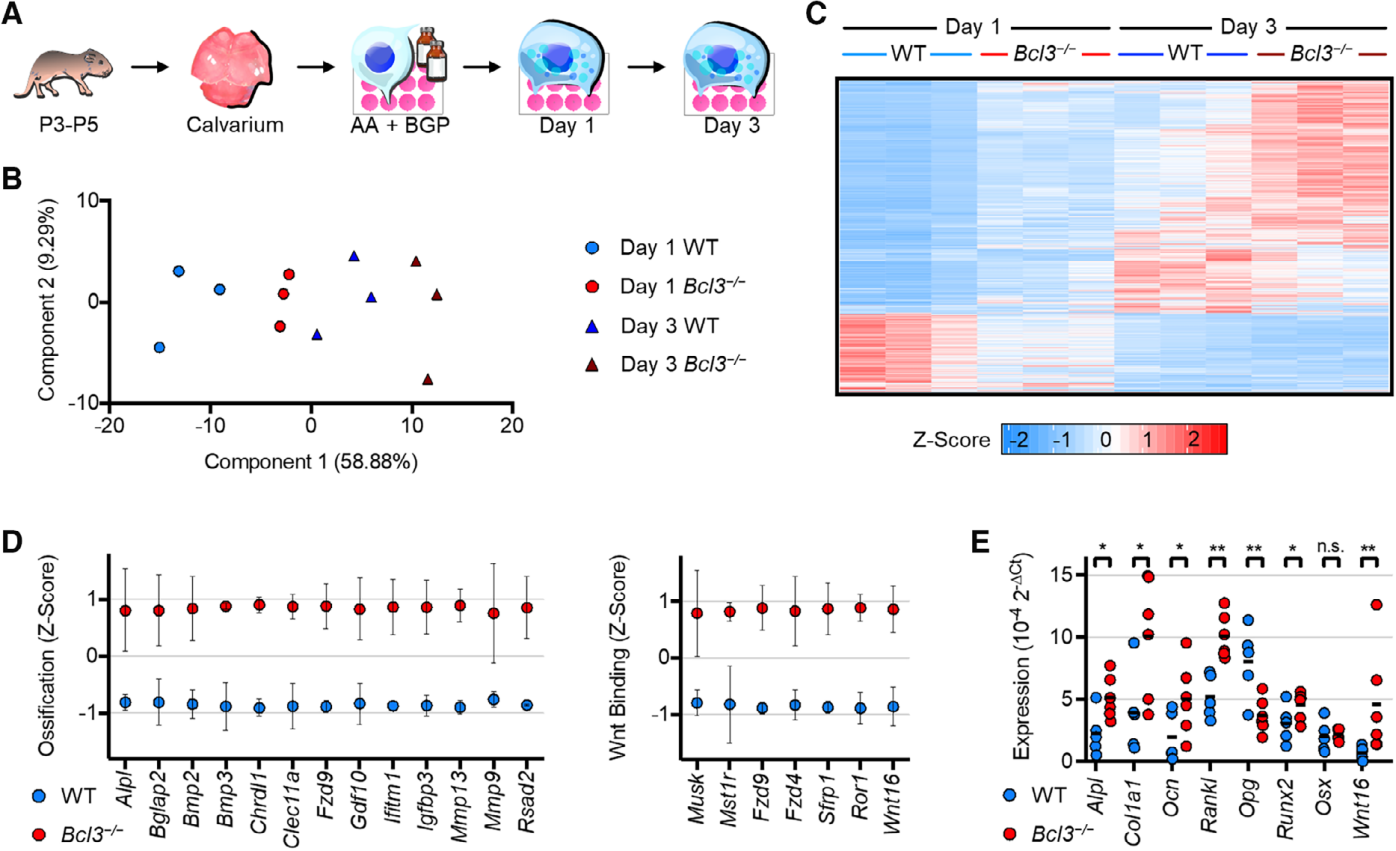
RNA sequencing transcriptomic profiling of wild‐type (WT) and *Bcl3* ^*−/−*^ osteoblasts on day 1 and day 3 following osteogenic induction. **A,** Schematic representation of experimental procedure. Following confluence, neonatal calvarial osteoblasts were treated with 50 μg/ml of L‐ascorbic acid (AA) and 2m*M* β‐glycerophosphate (BGP) to induce osteogenesis. **B,** Principal component analysis of all replicates along principal components 1 and 2. **C,** Heatmap of all replicates illustrating genes significantly different in WT and *Bcl3* ^*−/−*^ cells. Adjusted *P* < 0.05, n = 3. **D,** Mean expression per gene Z scores of ossification and Wnt‐protein binding/Wnt gene sets identified via gene ontology analysis of up‐regulated genes in *Bcl3* ^*−/−*^ cells on day 1. Error bars denote SD. **E,** Expression of select osteoblast genes on day 1 determined via quantitative polymerase chain reaction: alkaline phosphatase tissue‐nonspecific isozyme (*Alpl*), collagen type I a‐1 (*Col1a1*), osteocalcin (*Ocn*), *Rankl*, osteoprotegerin (*Opg*), Runt‐related transcription factor 2 (*Runx2*), osterix (*Osx*), and *Wnt16*. n = 5–6. DESeq2 outputs (**B** and **C**) and Fisher's least significant difference test (**E**) were used. n.s., not significant. * *P* < 0.05; ** *P* < 0.01.

To determine whether the increased bone density observed in *Bcl3* ^*−/−*^ neonates was a result of altered osteoblast differentiation and activity, the temporal transcriptional profile of WT and *Bcl3* ^*−/−*^ cells during osteogenic differentiation was evaluated. Calvarial osteoblast precursors were isolated from WT and *Bcl3* ^*−/−*^ neonates and differentiated in the presence of L‐ascorbic acid and β‐glycerophosphate for one or three days (Figure 2A). A principal component analysis of all genes across the two time points in WT and *Bcl3* ^*−/−*^ samples revealed that the bulk of variation (component 1) grouped the samples by genotype and time (Figure 2B). Hierarchical clustering of the significantly (false discovery rate–adjusted *P* < 0.05) up‐regulated and down‐regulated transcripts (Figure 2C and Supplementary Figure 1 and Supplementary Data, available at http://onlinelibrary.wiley.com/doi/10.1002/art.42639) confirmed this and revealed that at both time points, *Bcl3* ^*−/−*^ osteoblasts maintained an altered transcriptome relative to WT cells. To understand whether this represented an enhanced progression of *Bcl3* ^*−/−*^ cells through the osteogenic process, further analysis was performed to fully evaluate the altered transcriptional profiles. Initially, a comparison of the temporal (day 1 vs. day 3) difference (Δ) in fold change of all expressed genes between WT and *Bcl3* ^*−/−*^ cells revealed that transcriptional programs in both genotypes were highly correlated and shared a substantial proportion of significantly different genes (Supplementary Figure 1A and C, available at http://onlinelibrary.wiley.com/doi/10.1002/art.42639). These data demonstrate that both WT and *Bcl3* ^*−/−*^ cells share a very similar transcriptional program, namely osteogenesis.

The WT osteogenic program (ΔWT) was better correlated with, and shared a larger proportion of, significant genes between WT and *Bcl3* ^*−/−*^ cells on day 1 (Δ day 1) than on day 3 (Δ day 3; Supplementary Figure 1B, D, and E, available at http://onlinelibrary.wiley.com/doi/10.1002/art.42639). Collation of all genes up‐regulated or down‐regulated between WT and *Bcl3* ^*−/−*^ cells into a relative metagene reinforced the progressive osteogenic phenomenon and also highlighted the day 1 up‐regulated metagene in *Bcl3* ^*−/−*^ cells (Supplementary Figure 1F, available at http://onlinelibrary.wiley.com/doi/10.1002/art.42639). Comparison of the day 1 differentially up‐regulated genes in *Bcl3* ^*−/−*^ osteoblasts with the ΔWT signature differential genes on day 1 and day 3 revealed that *Bcl3* ^*−/−*^ day 1 cells were more similar to day 3 WT cells (228 overlapping transcripts) than to day 1 WT cells (one overlapping transcript; Supplementary Figure 1G, available at http://onlinelibrary.wiley.com/doi/10.1002/art.42639). Notably, some of the genes consistently up‐regulated in *Bcl3* ^*−/−*^ cells versus WT cells on day 1 and WT cells on day 3 were *Apoe*, *Col3a1*, *Col14a1*, *Chrdl1*, *Bglap2*, *Bmp2*, *Fbn2*, *Mmp3*, *Mmp9*, *Postn*, *Sfrp1*, *Stat1*, *Tmem178*, *Steap4*, *Wnt16*, and *Igfbp3* (Supplementary Data, available at http://onlinelibrary.wiley.com/doi/10.1002/art.42639). Taken together, this suggests that all cells are engaging in an osteogenic program, and already by day 1, *Bcl3* ^*−/−*^ cells are progressing through the process at an accelerated rate.

To further confirm the nature of the transcriptional program, GO analyses for broad biological process and specific molecular function were performed for the genes up‐regulated in *Bcl3* ^*−/−*^ cells on day 1 (Supplementary Figure 1H and I, available at http://onlinelibrary.wiley.com/doi/10.1002/art.42639). Notably, genes involved in extracellular matrix organization and ossification were among the most enriched processes (Supplementary Figure 1H, available at http://onlinelibrary.wiley.com/doi/10.1002/art.42639). Molecular function analysis revealed an enrichment of genes that participate in extracellular matrix interaction and modification, with gene products involved in Wnt‐protein binding being the most enriched set (Supplementary Figure 1I, available at http://onlinelibrary.wiley.com/doi/10.1002/art.42639). Classification of genes into the ossification gene set (most relevant to osteoblast function) and the most enriched Wnt‐protein binding gene set clearly illustrated the enhanced level of expression in *Bcl3* ^*−/−*^ cells (Figure 2D). Functional clustering of the 281 genes exclusively up‐regulated in *Bcl3* ^*−/−*^ cells on day 1 compared to WT cells revealed two gene subsets (Supplementary Figure 1J, available at http://onlinelibrary.wiley.com/doi/10.1002/art.42639). GO biological process/molecular function analyses identified one cluster surrounding the TNF and interleukin‐6 nodes with immune response/cytokine‐binding ontologies and another tight cluster with cell cycle/ATP‐binding ontologies. A transcription factor binding site analysis revealed upstream enrichment of known NF‐κB p65 sites and enrichment sites most similar to p50 and p52 binding motifs specific to the immune response gene cluster.

To validate the RNA sequencing findings, quantitative PCR was undertaken to evaluate osteoblast‐specific matrix encoding genes, including *Alpl*, *Col1a1*, and *Ocn*. Notably, these transcripts were significantly increased on day 1 in *Bcl3* ^*−/−*^ osteoblasts compared to WT controls (Figure 2E). Moreover, transcript levels of *Rankl* were higher and levels of *Opg* were lower in *Bcl3* ^*−/−*^ osteoblasts compared to WT controls (Figure 2E), suggesting an altered osteoblast activity that could increase early cross‐talk between osteoblasts and osteoclasts. The early‐stage Wnt‐driven prototypical osteoblast transcription factor, *Runx2*, also had elevated transcripts in *Bcl3* ^*−/−*^ osteoblasts compared to WT controls, whereas the later‐stage Wnt‐inhibiting transcription factor, osterix, had unchanged transcript levels (Figure 2E). Finally, expression of the ligand Wnt16 was confirmed to be elevated. Together, these data indicate that *Bcl3* ^*−/−*^ osteoblasts undergo an accelerated early osteogenesis program.

### BCL3 controls osteoblast differentiation

**Figure 3:**
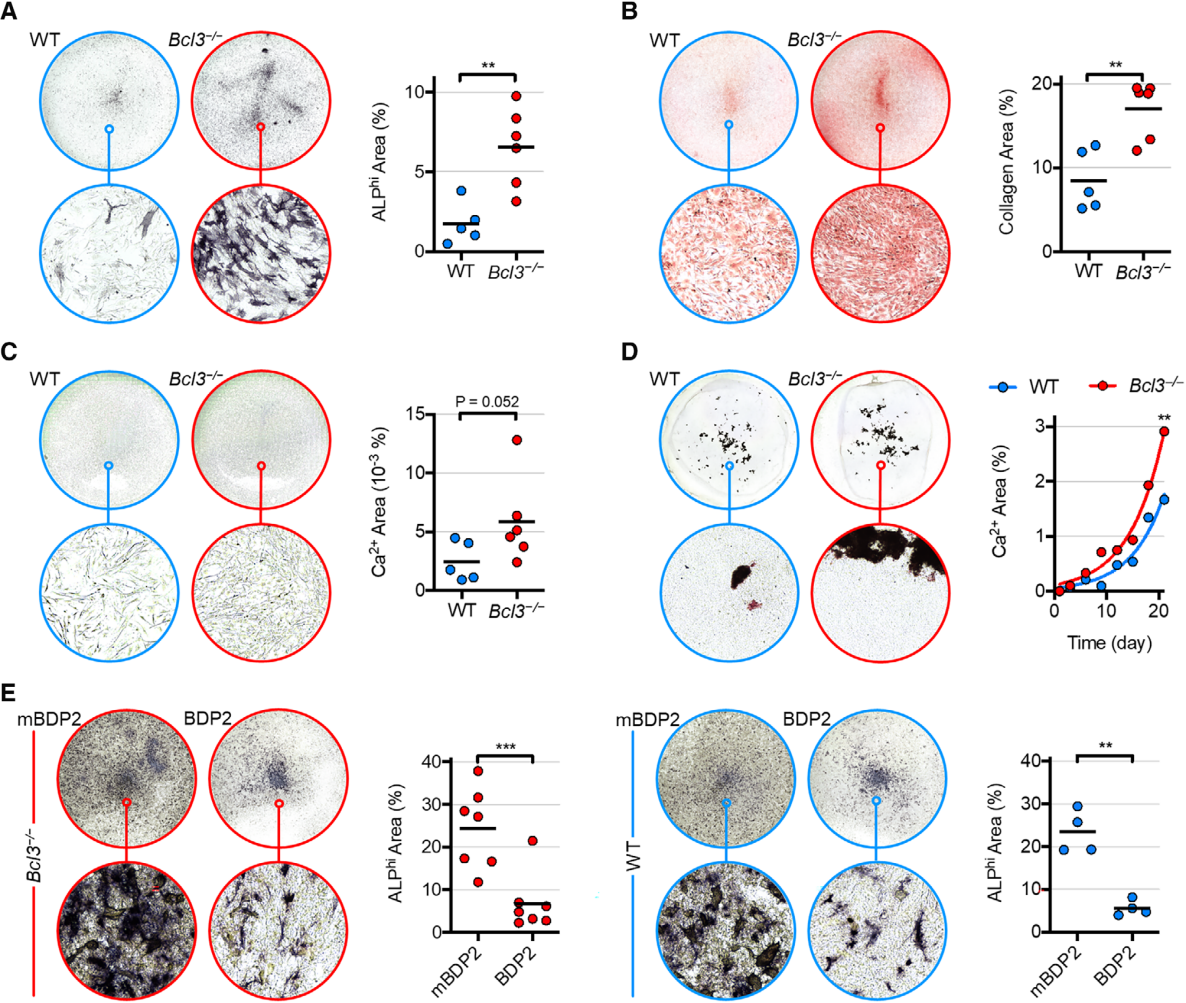
Characterization of wild‐type (WT) and *Bcl3* ^*−/−*^ osteoblast function following osteogenic induction and rescue/overexpression via BCL3 mimetic peptide. Osteogenesis was induced with 50 μg/ml of L‐ascorbic acid and 2 m*M* β‐glycerophosphate. **A**–**C,** Osteoblast production of alkaline phosphatase (ALP) (**A**), collagen (**B**), and calcium mineral micronodules (**C**) on day 1. Complete‐well representative images are of group medians (top; diameter = 22 mm) and the indicated magnified region (bottom; diameter = 182 μm), accompanied by pixel quantification. **C**, Enhanced sharpness and contrast in representative images. n = 5 (WT) and 6 (*Bcl3* ^*−/−*^). **D,** Calcium mineral nodule production until day 21, with mean values superimposed per 3‐day interval. The curve fitted to the exponential equation using least squares fit and means is shown. n = 40. **E,** ALP expression in WT and *Bcl3* ^*−/−*^ neonate calvarial osteoblasts under osteogenic differentiation until day 3 and treated with 30 *μM* control peptide (mutated BDP2) or BCL3 mimetic peptide (BDP2). Complete‐well representative images are of group medians (top; diameter = 15.6 mm) and the indicated magnified region (bottom; diameter = 182 μm), accompanied by pixel area quantification. n = 4–7. The Mann‐Whitney test (**A**–**C**), extra sum‐of‐squares F test (**D**), and paired *t*‐test (**E**) were used. * *P* < 0.05; ** *P* < 0.01; *** *P* < 0.001.

To investigate the functional consequences of the accelerated osteogenesis transcriptional program identified in *Bcl3* ^*−/−*^ preosteoblasts, in vitro differentiation assays were undertaken and cultures were characterized for mature osteoblast‐associated features. This included alkaline phosphatase (ALP), the prototypical osteoblast ectoenzyme that makes PO_4_ available for mineralization, which was markedly elevated in *Bcl3* ^*−/−*^ cultures compared to WT cultures on day 1 (Figure 3A) (25). Moreover, production of collagen (for extracellular secretion and incorporation into bone; Figure 3B) and calcium mineralization (microscopic bone nodules; Figure 3C) were also substantially increased in *Bcl3* ^*−/−*^ cultures compared to WT controls. Full characterization of calcified bone nodule formation to the point of osteoblast maturity (day 21) revealed a significantly increased cumulation of mineralized bone in *Bcl3* ^*−/−*^ cultures compared to WT controls (Figure 3D). Combined with our transcriptome analysis, these data demonstrate that lack of BCL3 enhances osteoblast differentiation and increases function.

We next investigated whether accelerated osteoblast differentiation and increased osteogenic function in cells deficient in BCL3 could be reversed by treating *Bcl3* ^*−/−*^ cells with a BCL3 mimetic peptide (BDP2) (26). Analysis of cultures on day 3 showed a significant decrease in ALP expression in the BDP2‐treated *Bcl3* ^*−/−*^ cultures compared to cells treated with an inactive control peptide (Figure 3E). Notably, similar results were obtained for BDP2‐treated WT cells (Figure 3E). Thus, a mimetic BCL3 peptide substantially restricts osteogenic differentiation, placing BCL3 as a strong regulator of osteogenesis.

### Osteoclastogenesis and resorption are controlled by BCL3

**Figure 4:**
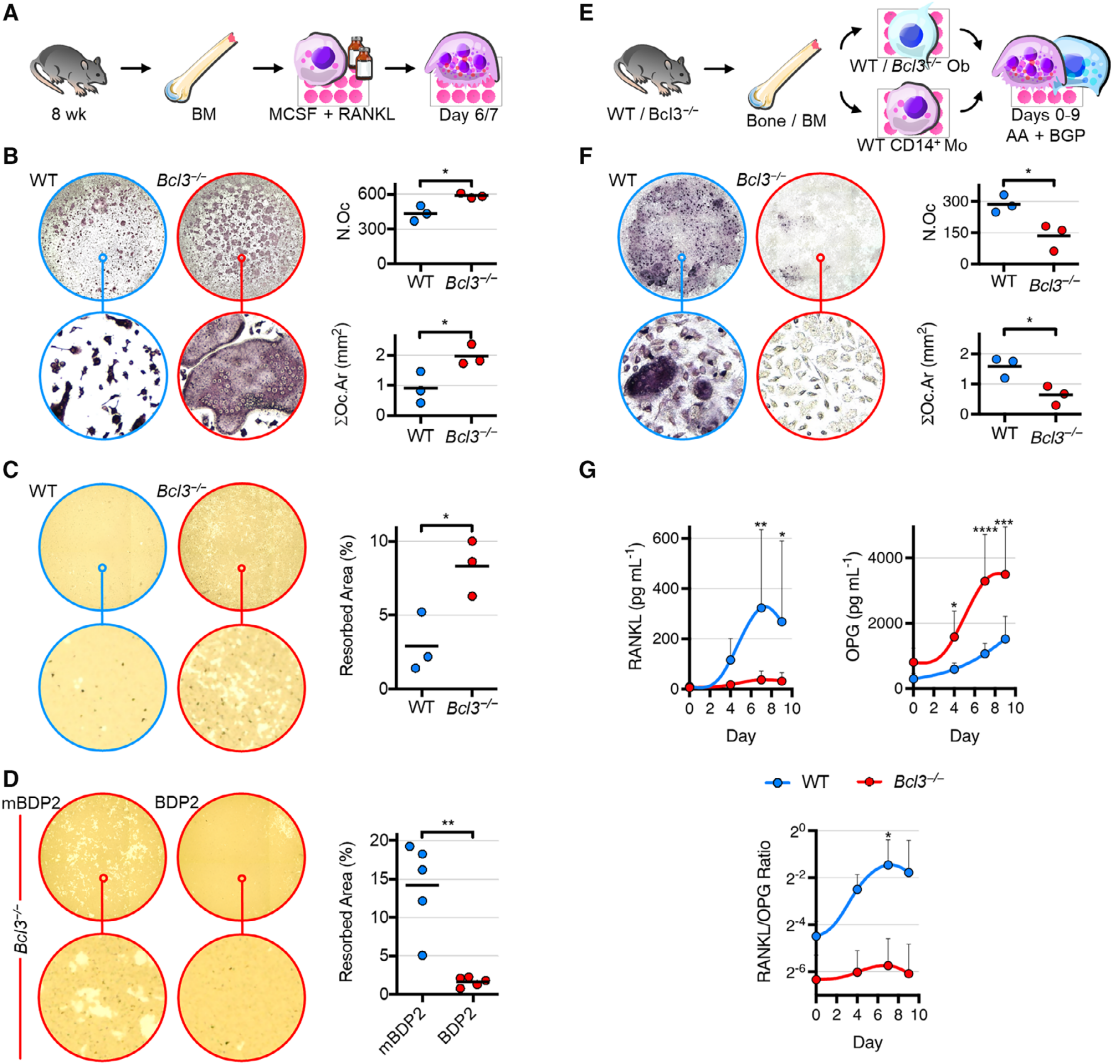
Characterization of osteoclastogenic differentiation and function of wild‐type (WT) and *Bcl3* ^*−/−*^ cells. **A,** Bone marrow (BM)–derived nonadherent cells were stimulated with 30 ng/ml of macrophage colony‐stimulating factor (M‐CSF) overnight and supplemented with 50 ng/ml of M‐CSF and RANKL until day 6 or 7. **B,** Tartrate‐resistant acid phosphatase (TRAP)–stained mature osteoclasts (≥3 nuclei) on day 6. N.Oc, osteoclast number; ∑Oc.Ar, total osteoclast area. n = 3. **C,** Resorption activity of osteoclasts cultured on mineral substrate until day 7. Lighter areas indicate resorbed mineral. n = 3. **D,** Resorption activity of *Bcl3* ^*−/−*^ osteoclasts under osteoclastogenic differentiation until day 7 and treated with 30 μ*M* control peptide (mutated BDP2) or BCL3 mimetic peptide (BDP2). n = 5. **E,** WT or *Bcl3* ^*−/−*^ (from a *Bcl3* ^*−/−*^ *::p53* ^*fl /−*^ strain*)* osteoblasts were cocultured with WT CD14+ monocytes in osteogenic medium up to 9 days. **F,** TRAP‐stained osteoclast number and area on day 9. n = 3. **G,** Supernatant levels of RANKL and osteoprotegerin (OPG). n = 5–6. Representative complete‐well (top; diameter = 6.4 mm) and magnified (bottom; diameter = 182 μm) images are of medians (**B**–**F**). Student's *t*‐test (**B–C** and **F**) and the paired *t*‐test (**D**) were used. Two‐way analysis of variance with Fisher's least significant difference multiple comparison (**G**) was used. * *P* < 0.05; ** *P* < 0.01; *** *P* < 0.001; **** *P* < 0.0001.

To assess whether the increase in skeletal bone density observed in neonatal *Bcl3* ^*−/−*^ mice was due exclusively to increased osteoblast activity or whether perturbation of intrinsic osteoclast activity might be involved, osteoclastogenesis was assessed. Adult mouse bone marrow myeloid cells were differentiated in vitro using M‐CSF and RANKL (Figure 4A). Evaluation of 6‐day differentiated cultures demonstrated that loss of BCL3 resulted in a significant increase in the number and size of mature osteoclasts compared to WT controls (Figure 4B). This also corresponded with an increase in resorptive activity (Figure 4C) in *Bcl3* ^*−/−*^ osteoclasts relative to WT cells. Notably, the increased resorptive activity of *Bcl3* ^*−/−*^ osteoclasts was suppressed by treatment with BDP2 peptide (Figure 4D).

To assess the impact of loss of BCL3 in the osteoblast lineage and how this subsequently alters osteoclastogenesis, osteoblast–osteoclast cocultures were conducted. Adult mouse WT CD14+ bone marrow–derived monocyte precursors were cultured with WT or *Bcl3* ^*−/−*^ long bone–derived mature osteoblasts for 9 days (Figure 4E). Assessment of osteoclastogenesis revealed that compared to WT controls, coculture with *Bcl3* ^*−/−*^ osteoblasts resulted in significantly smaller and fewer less mature osteoclasts (Figure 4F). Notably, this corresponded with significantly lower levels of RANKL but substantially higher levels of OPG in *Bcl3* ^*−/−*^ coculture supernatants relative to WT controls (Figure 4G). Moreover, the corresponding RANKL‐to‐OPG ratio was significantly increased for WT osteoblast cocultures (Figure 4G). Although *Bcl3* ^*−/−*^ osteoblasts did produce increased levels of *Wnt16* transcript and WNT16 protein compared to WT controls, levels in cocultures were not significantly increased, thus excluding differential WNT16 involvement in coupling with osteoclasts (Supplementary Figure 2B–D, available at http://onlinelibrary.wiley.com/doi/10.1002/art.42639). Evaluation of osteoclasts (cathepsin K+) in 12‐week‐old adult tibiae revealed that there was a significant decrease in osteoclast number per tissue area in *Bcl3* ^*−/−*^ mice compared to WT mice, whereas the number of osteoclasts per bone perimeter was unchanged, explained by the increased bone in *Bcl3* ^*−/−*^ sections (Supplementary Figure 2E, available at http://onlinelibrary.wiley.com/doi/10.1002/art.42639). Together, these data suggest that although BCL3 loss increases intrinsic osteoclastogenesis, it simultaneously enhances the ability of osteoblasts to restrict osteoclastogenesis, thus maintaining equilibrium.

### Adult mice lacking BCL3 have denser, stronger bones

**Figure 5:**
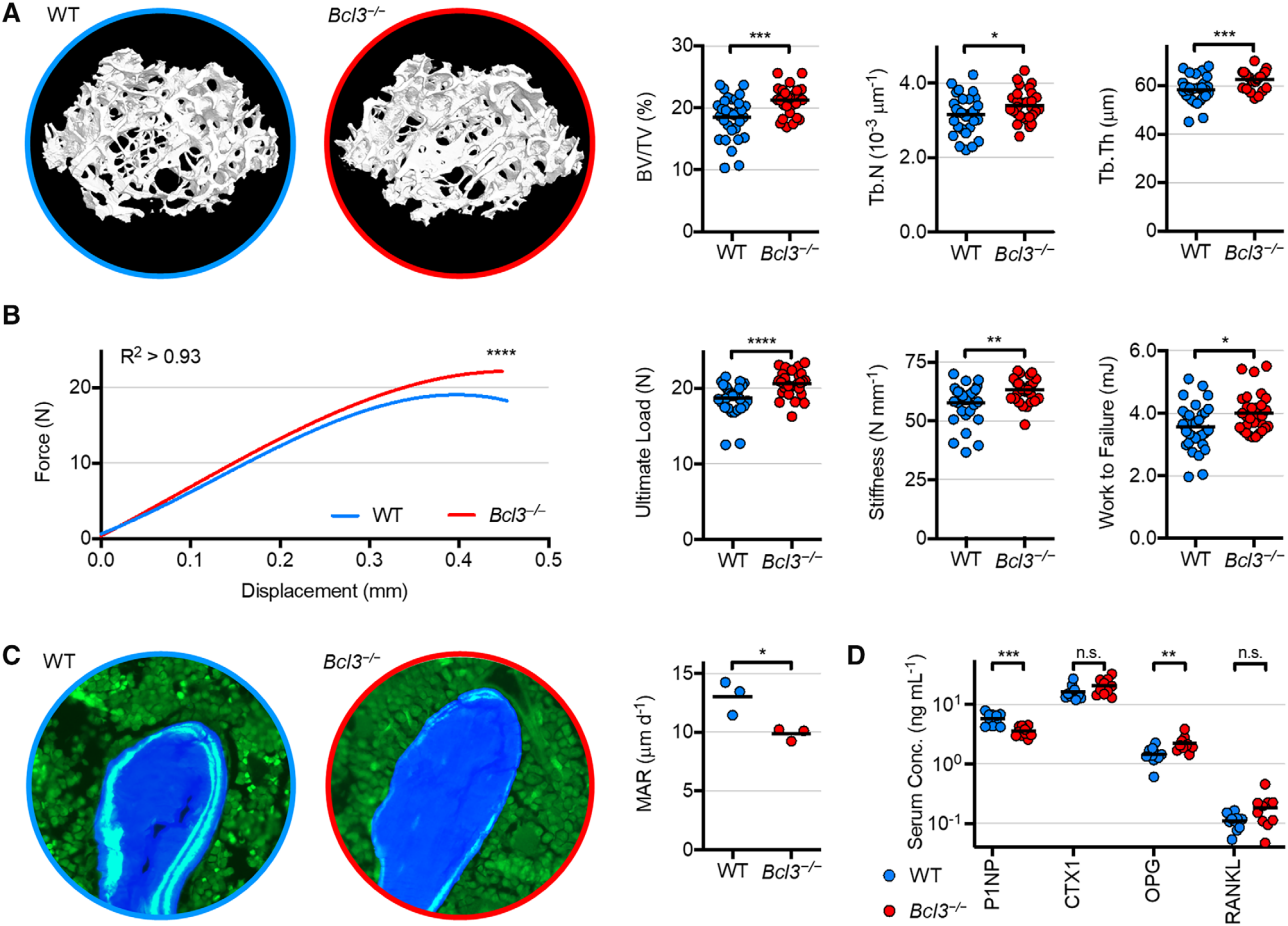
Phenotypic analyses of 20‐week‐old wild‐type (WT) and *Bcl3* ^*−/−*^ male mice. **A,** Representative volumetric bone density visualizations of the distal femoral trabecular region (images not to scale). Key three‐dimensional morphometric parameters: percent bone volume density (BV/TV), trabecular number (Tb.N), and trabecular thickness (Tb.Th). **B,** Biomechanical three‐point break test of the tibial mid‐shaft. The force‐displacement curve was fitted to the cubic equation using least squares fit. Derivative parameters include ultimate load (maximum), stiffness (linear slope), and work to failure (curve area). n = 29 (*Bcl3* ^*−/−*^) and 31 (WT). **C,** Dynamic histomorphometry of the combined trabecular and endocortical regions of the proximal metaphysis of tibiae. Representative images are of sections showing fluorescent calcein double label (light blue), bone matrix (dark blue), and bone marrow (green). MAR, mineral apposition rate. Image diameter = 190 μm. n = 3. **D,** Serum bone turnover markers procollagen type 1 amino‐terminal peptide (P1NP), carboxyl‐terminal crosslinks peptide of type 1 collagen (CTX1), osteoprotegerin (OPG), and RANKL. n = 10. Welch's *t*‐test or the Mann‐Whitney test was used for pairwise comparisons throughout, and the extra sum‐of‐squares F test was used (**B**). n.s., not significant. * *P* < 0.05; ** *P* < 0.01; *** *P* < 0.001; **** *P* < 0.0001.

Having identified increased skeletal mineral density in *Bcl3* ^*−/−*^ neonates and perturbation in both the osteoblast and osteoclast lineages, it was important to assess the impact on the adult skeleton. To achieve this, 20‐week‐old WT and *Bcl3* ^*−/−*^ male mice (which represent the peak adult stage of bone density, length, and thickness) were analyzed by micro‐CT (27). A morphometric analysis focused on the medullary trabecular regions of the distal femur and the proximal tibia and the distal cortical region of the femur (Figure 5A and Supplementary Figures [Link], [Link], available at http://onlinelibrary.wiley.com/doi/10.1002/art.42639). In both trabecular regions, percent bone volume density, trabecular number, and trabecular thickness were increased in *Bcl3* ^*−/−*^ mice compared to WT mice (Figure 5A and Supplementary Figures [Link], [Link], available at http://onlinelibrary.wiley.com/doi/10.1002/art.42639). Additionally, in *Bcl3* ^*−/−*^ mice, the bone surface‐to‐volume ratio and structural model index were significantly decreased in both trabecular regions, whereas only the tibial region showed increased tissue volume and degree of anisotropy, compared to in WT mice (Supplementary Figures 3 and 4B, available at http://onlinelibrary.wiley.com/doi/10.1002/art.42639). In the femoral cortical region, *Bcl3* ^*−/−*^ mice displayed a significantly increased mean total cross‐sectional bone area, mean total cross‐sectional tissue area, and cross‐sectional thickness compared to WT mice (Supplementary Figure 5A, available at http://onlinelibrary.wiley.com/doi/10.1002/art.42639). Furthermore, *Bcl3* ^*−/−*^ mice were also significantly different from WT mice in all other cortical morphometric parameters indicative of larger more stable cortical bone (Supplementary Figure 5B, available at http://onlinelibrary.wiley.com/doi/10.1002/art.42639).

To assess whether the increased bone density of *Bcl3* ^*−/−*^ male 20‐week‐old mice altered the biomechanical properties of load‐bearing bones, a three‐point break test of the tibial mid‐diaphysis was conducted. Force‐displacement curves for *Bcl3* ^*−/−*^ mice were significantly different from those for WT mice (Figure 5B) and were used to derive key biomechanical parameters. Essentially, *Bcl3* ^*−/−*^ tibiae had significantly increased ultimate load, stiffness, and work to failure when compared to WT tibiae, indicative of increased biomechanical strength (Figure 5B). The femoral neck break test was also performed to support these findings and further demonstrated that *Bcl3* ^*−/−*^ bones had increased biomechanical strength, as defined by ultimate load and stiffness (Supplementary Figure 6, available at http://onlinelibrary.wiley.com/doi/10.1002/art.42639).

To investigate new bone mineralization in the adult (20‐week‐old) skeleton, dynamic histomorphometry of femoral sections from mice administered with calcein double label was assessed. Analysis of femurs showed that by 20 weeks, *Bcl3* ^*−/−*^ mice had a decreased mineral apposition rate (MAR) when compared to WT controls (Figure 5C). To further explore bone turnover, serum markers P1NP (associated with bone formation) and CTX1 (associated with bone resorption) were evaluated. The levels of P1NP were significantly lower in the serum of *Bcl3* ^*−/−*^ mice compared to WT controls, whereas the levels of CTX1 were not significantly different from WT mice (Figure 5D). Serum levels of the osteoblast–osteoclast coupling factor, RANKL, were unchanged, but levels of its decoy competitor, OPG, were significantly increased in 20‐week‐old *Bcl3* ^*−/−*^ mice compared to WT mice (Figure 5D); however, the RANKL‐to‐OPG ratio was unchanged (*P* = 0.04, median of differences = 0.02, Wilcoxon test). Combined, this suggests that adult *Bcl3* ^*−/−*^ mice maintain increased bone density and strength, but the osteoblast‐driven turnover‐to‐mineralization rate of bone decelerates by 20 weeks of age.

### Absence of BCL3 restricts pathological osteophytic formation

**Figure 6:**
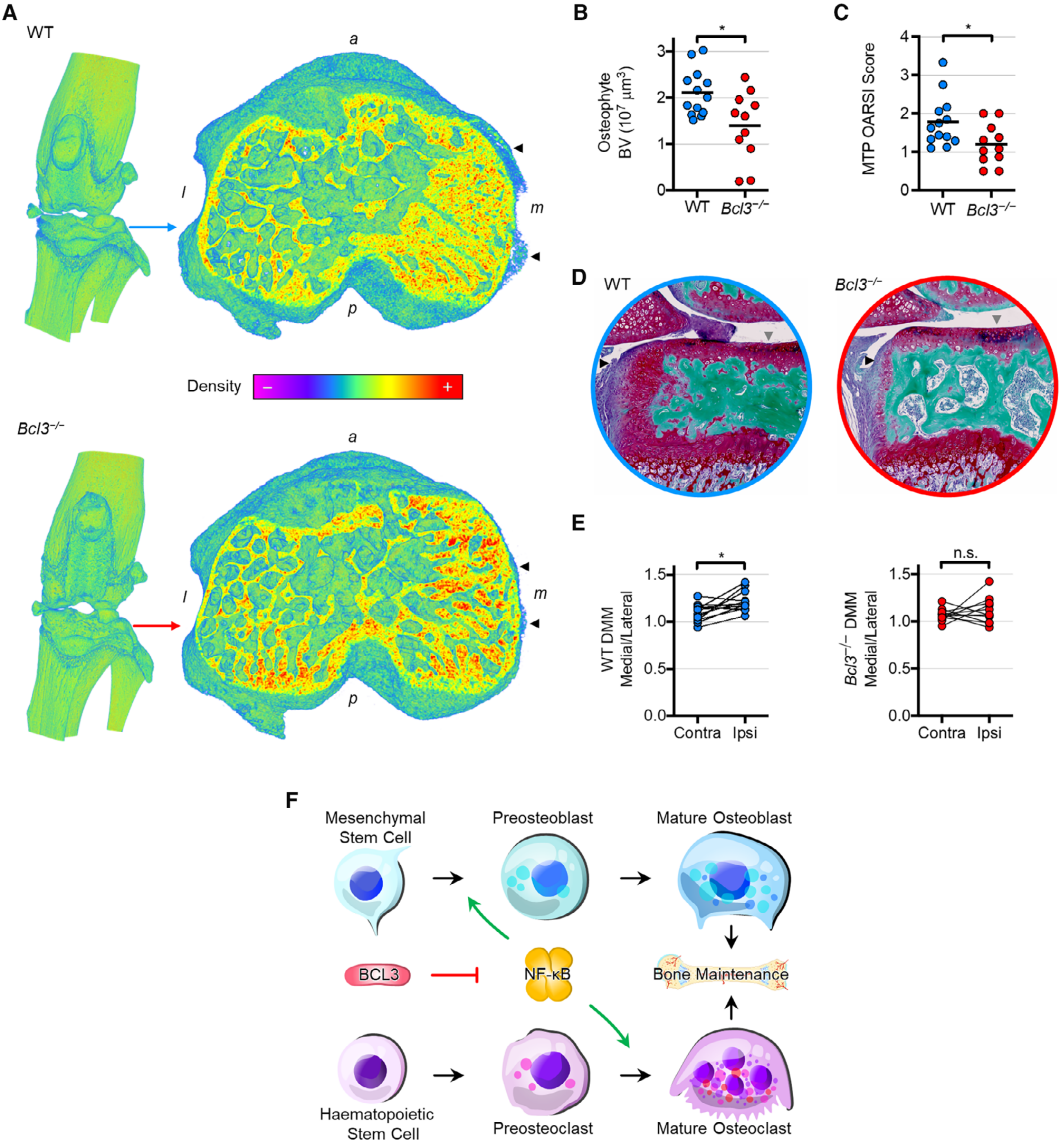
Osteoarthritic disease model of the destabilized medial meniscus (DMM) in wild‐type (WT) and *Bcl3* ^*−/−*^ mice. **A,** Knee joint (left) and cross‐section of the tibial subchondral bone plateau (right) illustrating the protrusion of pathologic osteophytes (arrowheads) 14 days post surgery. Representative images are shown in the bone density spectrum. *a*, anterior; *l*, lateral; *m*, medial; *p*, posterior. **B,** Total bone volume (BV) of osteophytes in WT and *Bcl3* ^*−/−*^ mice subjected to DMM procedure. **C,** Osteoarthritis Research Society International (OARSI) histopathology scoring of the medial tibial plateau (MTP) of WT and *Bcl3* ^*−/−*^ mice 14 days post surgery. **D,** Dual‐stained sections of identical WT and *Bcl3* ^*−/−*^ joint samples in **A** showing red subchondral osteophyte (black arrowheads) and hairy articular cartilage damage (grey arrowheads). Image diameter = 1 mm. **E,** Tibial subchondral bone medial‐to‐lateral bone volume ratios in contralateral (unoperated) and ipsilateral (operated) knees. Welch's *t*‐test (**B** and **C**) and the ratio paired *t*‐test (**E**) were used. n = 11 (*Bcl3* ^*−/−*^) and 13 (WT). n.s., not significant. * *P* < 0.05. **F,** Schema of perturbed skeletal steady state in mice lacking BCL3. Homeostatic progression of both osteogenesis and osteoclastogenesis is dependent on NF‐κB activity and regulated by BCL3. Herein, we have shown that BCL3 absence accelerates differentiation and increases function of both osteoblasts and osteoclasts.

Given the accelerated osteogenesis in neonates and the increased bone density in adults mixed with a decrease in bone formation in *Bcl3* ^*−/−*^ mice, it was important to assess the in vivo mineralization of new bone in adults. To achieve this, *Bcl3* ^*−/−*^ and WT mice were subjected to DMM, which leads to the generation of new ectopic mineralization in the form of osteophytes (24). Fourteen days post surgery, the micro‐CT analysis revealed that in the affected medial region of the tibial subchondral bone, protruding mineralized osteophyte projections were visibly reduced in *Bcl3* ^*−/−*^ mice compared to WT controls (Figure 6A). Quantification of the total osteophyte bone volume showed that *Bcl3* ^*−/−*^ mice had significantly lower osteophyte bone volume when compared to WT counterparts (Figure 6B). Histopathological scoring of articular cartilage at the medial tibial plateau showed that compared to WT mice, *Bcl3* ^*−/−*^ mice had a lower deterioration (Figure 6C and D). The histological evaluation also revealed that pathological articular cartilage fibrillation and cartilaginous osteophyte extension were present in WT mice but reduced in *Bcl3* ^*−/−*^ mice (Figure 6D). Notably, unlike WT mice, *Bcl3* ^*−/−*^ mice did not have a significantly increased subchondral osteosclerosis in the bone loading regions of the tibial plateau of DMM‐induced knees compared to contralateral controls, indicative of reduced disease (Figure 6E and Supplementary Figure 7, available at http://onlinelibrary.wiley.com/doi/10.1002/art.42639).

Taken together, these data demonstrate that loss of BCL3 inhibits pathologically driven new bone formation/mineralization in adults while maintaining increased congenic global bone density.

## DISCUSSION

The tightly regulated equilibrium between osteoblasts and osteoclasts determines skeletal development and maintenance. The master transcription factor, NF‐κB, plays a key role in the differentiation of both cell types and is thus crucial for maintaining skeletal health (4). In the present study, we have shown that BCL3, a known regulator of NF‐κB, has an integral role in modulating skeletal development and maintenance. Assessment of neonates clearly demonstrated that loss of BCL3 led to an increase in bone density that was coupled with impeded long bone elongation. A transcriptomic analysis revealed that this was associated with an accelerated differentiation program in *Bcl3* ^*−/−*^ osteoblast precursors that drove increased osteogenesis, which ultimately resulted in increased bone matrix production. Intrinsic osteoclastogenesis was shown to be regulated by BCL3 and ultimately restricted by osteoblast coupling through the RANKL–OPG axis. Surprisingly, although the increased bone density (and associated superior biomechanical strength) in *Bcl3* ^*−/−*^ mice was maintained into adulthood, osteoblast‐driven generation of new bone was substantially decreased. This adult‐specific phenotype was also associated with suppression of osteosclerosis and osteophytogenesis in the DMM model of osteoarthritic disease.

The altered bone biology observed in *Bcl3* ^*−/−*^ mice is reflective of the major role BCL3 plays in the regulation, rather than complete control, of NF‐κB family members (5). This is illustrated by the more profound bone phenotypes seen in other NF‐κB family member knockouts. For instance, double deletion of p50 and p52, both of which interact with BCL3 (5), results in dwarfism and severe osteopetrosis (16). The regulatory nature of BCL3 is further supported by prior studies showing that BCL3 is key for the maintenance of murine embryonic stem cell pluripotency and subsequent differentiation, with overexpression substantially attenuating but not blocking cell differentiation (28) and overexpression of BCL3 in the myeloid lineage reducing but not completely blocking RANKL‐driven osteoclastogenesis (29). Both observations are analogous to those seen in the in vitro osteoblastogenesis and osteoclastogenesis mimetic peptide cultures (Figures 3 and 4). Combined and added to the observation that the transcriptional program in the osteoblast lineage is accelerated (Figure 2), this supports the concept that BCL3 acts as a negative regulator of the critical NF‐κB machinery involved in the differentiation programs of multiple skeletal cell types.

In the mesenchymal cell lineage, BCL3 has recently been shown to regulate cross‐talk between Wnt signaling pathway components (30). Moreover, NF‐κB p65 activation (driven by low‐dose TNF), with subsequent signaling through the Wnt pathway, substantially increases osteogenic function (14). It is therefore noteworthy that the accelerated transcriptional response in *Bcl3* ^*−/−*^ preosteoblasts included enhancement of genes involved in Wnt regulation, in particular increased expression of *Wnt16*, which provides a mechanism linking transient NF‐κB activity to enhanced subsequent osteogenic activity (31, 32, 33). Interestingly, mutations in *Wnt16* were recently identified as highly significantly associated with risk of fracture, whereas Wnt16 was found to inhibit chondrocyte hypertrophy and be protective against osteoarthritis progression (34, 35, 36). We showed increased expression of both *Wnt16* transcript and WNT16 protein in *Bcl3* ^*−/−*^ osteoblasts. Furthermore, the transcription factor signature of *Bcl3* ^*−/−*^ cells revealed a propensity for increased NF‐κB p65, p50, and p52 transcriptional activation alongside altered cell cycle and energetics‐associated transcription factors. The transcription factors PU.1, interferon regulatory factors, and STATs, evidently involved in early osteogenesis in both WT and *Bcl3* ^*−/−*^ cells, are known to cooperate with each other, especially in the immune cells in which BCL3 is critical alongside NF‐κB at gene promoters in a myriad of ways (37).

Increased osteogenic mineralization in early development, followed by reduced osteoblast‐driven bone turnover, in *Bcl3* ^*−/−*^ mice is a model analogous to the increased mineralization occurring in WT male mice versus female mice, in which the initial MAR at 1 week of age is lower in females but exceeds that of males by 20 weeks of age (27). Thus, increased density and strength concurrent with reduced bone formation (decreased P1NP and MAR) by 20 weeks is further evidence of accelerated skeletal maturation and an altered steady state in adulthood. *Bcl3* ^*−/−*^ osteoblasts in cocultures regulate osteoclast differentiation by tipping the RANKL‐to‐OPG ratio in favor of reduced osteoclastogenesis, indicating an inhibition of bone resorption. The decreased bone formation along with the reduction of osteoclast differentiation depicts an adult bone with reduced bone remodeling in the absence of BCL3, which ultimately results in maintenance of developmental‐driven bone mass.

The decrease in osteophytogenesis we observed in *Bcl3* ^*−/−*^ mice links to the tempered ability to generate new mineralized bone in adults. Osteophyte formation in the DMM model is associated with a characteristic chondrocytic core, which leads to progressive mineralization, in a process reminiscent of endochondral ossification (24). We posit that in *Bcl3* ^*−/−*^ mice, the chondrocyte–osteoblast lineage favors osteoblastic differentiation, impeding endochondral bone elongation in neonates and leading to a reduced chondrocytic core and suppressing the formation of pathology‐driven osteophytes in adults. Simultaneously, the osteophyte model functions to affirm the inverse relationship between increased bone density and endochondral protrusion (27, 38). The subdued effect on subchondral bone change in DMM‐induced *Bcl3* ^*−/−*^ mice is coupled with the reduction of osteophytogenesis as a pathological indicator and likely involves the same osteochondral lineage dynamics that are causal to the increased bone density in *Bcl3* ^*−/−*^ mice.

One of the limitations of our study was that we did not investigate osteochondroprogenitor differentiation into chondrocytes or the process of chondrocyte‐driven endochondral ossification (39, 40, 41). Chondrogenic differentiation, proliferation, and hypertrophy is controlled by NF‐κB subunit p65 activity (42, 43). Nevertheless, recent work supports our model and provides evidence for the role of BCL3 in chondrocytic callus‐driven fracture healing, in which *Bcl3* ^*−/−*^ mice exhibit delayed chondrocyte hypertrophy that is rescued with BCL3 overexpression (44). It is interesting to speculate therefore, given the inverse role of NF‐κB during osteoblast and chondrocyte differentiation (4), that loss of BCL3 fundamentally pivots NF‐κB such that differentiation of osteochondroprogenitors favors the osteoblast lineage. To fully interrogate this hypothesis, additional studies using conditional *Bcl3* ^*−/−*^ mice are required.

In summary, our study definitively shows that BCL3 plays a major role in the control of osteoblast and osteoclast differentiation and cross‐talk, which fundamentally regulates bone turnover and skeletal maintenance (Figure 6F). Beyond elucidating BCL3 function, this has implications for furthering understanding of NF‐κB in the skeleton and for possible therapeutic targets in skeletal pathologies.

## AUTHOR CONTRIBUTIONS

All authors were involved in drafting the article or revising it critically for important intellectual content, and all authors approved the final version to be published. Prof. Goodyear had full access to all of the data in the study and takes responsibility for the integrity of the data and the accuracy of the data analysis.

### Study conception and design

Jaffery, Carmody, Goodyear.

### Acquisition of data

Jaffery, Huesa, Chilaka, Cole, Doonan, Akbar, Dunning.

### Analysis and interpretation of data

Jaffery, Huesa, Tanner, van ‘t Hof, McInnes, Carmody, Goodyear.

## Supporting information


Disclosure form

**Appendix S1:** Supplementary Online Materials and Methods

**Data S1:** Supplementary Data

**Supplementary Figure 1:** RNA‐seq transcriptomic profiling of WT and *Bcl3* ^*−/−*^ osteoblasts at day 1 and day 3 following osteogenic induction. **a,** Significantly (*P*_*adj*_ < 0.05) differentially expressed genes between day 1 and day 3, with > 1 absolute fold‐change (FC) in both WT and *Bcl3* ^*−/−*^ cells. Venn diagrams illustrate shared or differentially expressed genes in both genotypes at day 1 and day 3. **b,** Significantly (*P*_*adj*_ < 0.05) differentially expressed genes in *Bcl3* ^*−/−*^ cells, relative to WT, with > 1 absolute fold‐change (FC) at day 1 and day 3. Venn diagrams illustrate sustained or transient upregulated and downregulated genes in *Bcl3* ^*−/−*^ cells. **c**‐**e,** Correlations of gene expression fold‐changes (FC) through time, from day 1 to day 3, in WT (Δ WT) and *Bcl3* ^*−/−*^ (Δ *Bcl3* ^*−/−*^) genotypes during osteogenesis (**c**), between Δ WT and *Bcl3* ^*−/−*^ genes at day 1 (Δ Day 1) compared to WT controls (**d**), and between Δ WT and *Bcl3* ^*−/−*^ genes at day 3 (Δ Day 3) compared to WT controls (**e**). Venn diagrams to the right of each panel indicate significantly different genes (*P*_adj_ < 0.05, fold‐change ± 1) to corresponding datasets on each axis. Spearman's rank correlation coefficient, r. n = 14493. **f,** Median expression level Z‐scores of upregulated (left) and downregulated (right) gene aggregates (metagene) in experimental replicates, relative to the gene expression change in WT cells between days 1 and 3 (Δ WT). n = 3. Student's *t*‐test for pairwise comparisons. **g,** Genes upregulated in *Bcl3* ^*−/−*^ cells at day 1 (light red circle), compared to genes differentially expressed between day 1 (light blue circle) and day 3 (dark blue circle) in WT cells. Overlapping regions represent shared gene‐sets. **h**‐**i,** Gene ontology (GO) analysis of biological process (**h**) and molecular function (**i**) of genes upregulated in *Bcl3* ^*−/−*^ cells at day 1, compared to WT. The top seven results in order of fold enrichment are highlighted. **j,** Genes significantly upregulated at day 1, exclusively in *Bcl3* ^*−/−*^ cells (‘281’ transcripts from **a**), subjected to protein‐protein association functional clustering analysis. Two major clusters with distinct top gene ontology‐determined biological process and molecular function are shown: ‘immune response’ / ‘cytokine binding’ and ‘cell cycle’ / ‘ATP binding’. Enriched transcription factor (TF) binding site TFBS analysis, for each cluster, displaying the top seven hits ordered by increasing *P*‐value of known TF motif and the *de novo* motifs of uniquely identified sites with the predicted transcription factor. * *P* < 0.05, *** *P* < 0.001 and **** *P* < 0.0001.

**Supplementary Figure 2:** Characterisation of *Bcl3* and *Wnt16* expression in osteoblasts, WNT16 protein in osteoblast‐osteoclast co‐cultures and osteoclast activity in adult bone of WT and *Bcl3* ^*−/−*^ mice. **a,** *Bcl3* gene expression in control and ascorbic acid (AA) stimulated murine MC3T3‐E1 osteoblast cells at day 5 (GEO accession: GSE37676). n = 3. **b,** *Bcl3* and *Wnt16* transcript expression (RNA‐seq mapped) in WT and *Bcl3* ^*−/−*^ osteoblasts at days 1 and 3. n = 3. **c,** WNT16 protein levels in osteoblast culture supernatants at day 9, without monocytes/osteoclasts. n = 5‐6. **d,** WNT16 levels in osteoblast‐osteoclast co‐culture supernatants until day 9. n = 5‐6. **e,** Representative images and quantification of cathepsin K (CTSK) stained sections, in brown, of tibiae from adult 12‐week WT and *Bcl3* ^*−/−*^ mice. Image diameter = 400μm. Number of osteoclasts (N.Oc) per tissue area (T.Ar) and number of osteoclasts per bone perimeter (B.Pm). n = 4‐5. Student's *t*‐test (**a**, **c** and **e**) or Two‐way ANOVA (**b** and **d**). * *P* < 0.05, ** *P* < 0.01, **** *P* < 0.0001 and ‘n.s.’ ‐ not significant.

**Supplementary Figure 3:** Additional parameters of the femoral trabecular region of 20‐week WT and *Bcl3* ^*−/−*^ male mice, including tissue volume (TV), bone volume (BV), bone surface (BS), bone surface density (BS/TV), bone surface/volume ratio (BS/BV), connectivity density (Conn.Dn), structural model index (SMI), trabecular separation (Tb.Sp), standard deviation of trabecular thickness [SD(Tb.Th)], standard deviation of trabecular separation [SD(Tb.Sp)], degree of anisotropy (DA) trabecular pattern factor (Tb.Pf). n = 29‐31. Welch's *t*‐test or Mann‐Whitney test. ** *P* < 0.01, *** *P* < 0.001, **** *P* < 0.0001 and ‘n.s.’ ‐ not significant.

**Supplementary Figure 4:** Phenotypic analyses of 20‐week WT and *Bcl3* ^*−/−*^ male mice proximal tibial trabecular region**. a,** Representative volumetric bone density visualisations (images not to scale) and key three‐dimensional morphometric parameters, including percent bone volume density (BV/TV), trabecular number (Tb.N), and trabecular thickness (Tb.Th). **b,** Additional parameters of the tibial trabecular region of 20‐week WT and *Bcl3* ^*−/−*^ male mice, including tissue volume (TV), bone volume (BV), bone surface (BS), bone surface density (BS/TV), bone surface/volume ratio (BS/BV), connectivity density (Conn.Dn), structural model index (SMI), trabecular separation (Tb.Sp), standard deviation of trabecular thickness [SD(Tb.Th)], standard deviation of trabecular separation [SD(Tb.Sp)], degree of anisotropy (DA) trabecular pattern factor (Tb.Pf). n = 29‐31. Welch's *t*‐test or Mann‐Whitney test. * *P* < 0.05, ** *P* < 0.01, *** *P* < 0.001, **** *P* < 0.0001 and ‘n.s.’ ‐ not significant.

**Supplementary Figure 5:** Phenotypic analyses of 20‐week WT and *Bcl3* ^*−/−*^ male mice distal femoral cortical region. **a,** Representative volumetric bone density visualisations (images not to scale) and key two‐dimensional parameters, including mean total cross‐sectional bone area (B.Ar), mean total cross‐sectional tissue area (T.Ar) and cross‐sectional thickness (Cs.Th). **b,** Additional parameters of the femoral cortical region of 20‐week WT and *Bcl3* ^*−/−*^ male mice, including cortical area fraction (B.Ar/T.Ar), tissue volume (TV), bone volume (BV), percent bone volume (BV/TV), tissue surface (TS), bone surface (BS), bone surface/volume ratio (BS/BV), mean total cross‐sectional tissue perimeter (T.Pm), mean total cross‐sectional bone perimeter (B.Pm), peripheral tissue surface [TS(per)], peripheral bone surface [BS(per)], mean polar moment of inertia [MMI(polar)], average object equivalent circle diameter per slice (Av.Obj.ECDa), average principal moment of inertia minimum [Av.MMI(min)], average principal moment of inertia maximum [Av.MMI(max)] and eccentricity (Ecc). n = 29‐31. Welch's *t*‐test or Mann‐Whitney test. * *P* < 0.05, ** *P* < 0.01, *** *P* < 0.001 and ‘n.s.’ ‐ not significant.

**Supplementary Figure 6:** Biomechanical outputs of the femoral neck break test of 20‐week WT and *Bcl3* ^*−/−*^ male mice. n = 14. Welch's *t*‐test or Mann‐Whitney test. * *P* < 0.05 and ‘n.s.’ ‐ not significant.

**Supplementary Figure 7:** Characterisation of the destabilisation of the medial meniscus (DMM) model of osteoarthritic disease progression in WT and *Bcl3* ^*−/−*^ in male mice, 2 weeks post‐procedure (12‐week age). Mice in the ‘Sham’ groups, which did not receive a medial meniscotibial ligament (MMTL) transection. The ratio of medial to lateral cross‐sectional percent bone volume (BV/TV) of the tibial plateau – between the ipsilateral (operated) and contralateral (unoperated) knees – the disparity representing a measure of disease via differential loading. n = 9 (*Bcl3* ^*−/−*^) and 8 (WT). Ratio paired *t*‐test. ‘n.s.’ ‐ not significant.
