# Supplementary material for: IĸB Protein BCL3 as a Controller of Osteogenesis and Bone Health
Source: Arthritis Rheumatol. 2023 Oct 1;75(12):2148–60. doi: 10.1002/art.42639 (PMC10952620; doi:10.1002/art.42639)
Supplement: Supplementary file 2 — Appendix S1: Supplementary Online Materials and Methods [file ART-75-2148-s008.docx]

The IĸB protein BCL3 controls osteogenesis and bone health

Jaffery *et al*

**Supplementary Online Materials and Methods**

**Animals.**

A single animal constituted the experimental unit in statistical comparisons; except for neonatal calvarial cultures, where 3 pooled mice constituted a single experimental replicate. For calculation of the experimental sample size number (n) in micro-CT experiments, statistical *a priori* power prediction was constrained to the following parameters: normal distribution, power (1-β error probability) = 0.99, α error probability = 0.01, allocation ratio n_2_/n_1_ = 1, effect sizes (d) of 1.35 and 2.5 based on resolving a range of primary outcome micro-CT morphometric parameters (e.g., BV/TV, Tb.N and Tb.Th), in a pilot study, and Wilcoxon-Mann-Whitney two-tailed *t*‑tests for two groups. Between genotypes, a simultaneous age-matched timed-mating procedure was arranged. Intra-genotype allocation to control and treatment groups, specifically for DMM surgical model, was conducted using a randomly alternating/matching sequence and confounding was avoided by maintaining sham control littermates as cagemates. Global confounding was avoided by maintaining identical environmental, handling, procedural and sample collection techniques, across age-matched genotype controls (*Bcl3^−/−^* and WT) that were bred and reared simultaneously. Animals were assigned numerical identifiers at procedural initiation by the primary experimenter to provide blinding from group-wise information during the complete process of sample collection, processing and especially data analysis.

For the *ex vivo* micro-CT experiment comparing 29 *Bcl3^−/−^* and 31 WT adult mice, a single definitive experimental outlier in the *Bcl3^−/−^* group was found during analysis of multiple parameters using the *a priori*-determined ROUT method (Q = 0.1%) and was excluded from further analysis based on its gross physiological anomaly. No other experiments had any identified outliers.

**Micro-CT analysis of neonates.**

Euthanised neonatal (post-natal day 0) mice were immersed in 4% (w/v) formaldehyde in DPBS (Dubecco’s phosphate-buffered saline) overnight, then stored in 70% EtOH at 4°C. Pups were maintained in 70% EtOH during micro-CT scanning. Images had a resolution of 4.5 μm and were acquired using automatic stitching. Prior to reconstruction, the image dataset was compressed to give an effective resolution of 20 μm. Reconstructions were manually analysed using DataViewer (version 1.5.2.4) to measure the lengths of the long bones. Whole-specimen bone mineral density (BMD) analysis was conducted, using two hydroxyapatite epoxy resin phantoms of known mineral densities to calibrate attenuation coefficients and a voxel intensity range between 15 to 255 was selected. Visualisations were rendered in CTvox (version 3.1.1) and calcified mineral was assigned pseudocolour of a spectrum ranging from red (high voxel intensity) to violet (low voxel intensity).

**Whole-mount double-staining of neonates.**

Euthanised neonatal (post-natal day 0) mice were scalded by immersion in dH_2_O at 65**°**C for 30s, to allow maceration of tissue and skin removal. After removal of soft tissues including skin, eyes, internal organs and adipose tissue, mice were fixed in 95% EtOH overnight, then immersed in 100% acetone overnight. Cartilage was stained by submerging mice overnight in filtered Alcian blue stain solution (0.03% (w/v) Alcian Blue 8GX, 80% EtOH, 20% glacial acetic acid) (1). Destaining was conducted by washing neonates thrice in 70% EtOH for 5 min and incubating in 95% EtOH overnight. Tissue was then pre-cleared in 1% (w/v) KOH for 1h. Calcium mineral was stained with Alizarin Red solution (0.005% (w/v) Alizarin Red S solute in 1% (w/v) KOH solvent) at 4**°**C overnight (1). Destaining and clearing of tissue was achieved by submersion in a clearing solution (50% glycerol and 50% of 1% (w/v) KOH) until judged to be destained. Mice were stored in 100% glycerol and imaged with light microscopy. Tibia colour analysis using the program Fiji/ImageJ (version 2.0) involved specification of a region of interest at the epiphysis of the tibia, splitting of red-green-blue colour channels and obtaining the mean grey value for red (positive) and green (background) channels.

**Calvarial osteoblast isolation.**

Euthanised neonatal mice (post-natal days 3 to 5) were decapitated, calvaria were dissected in DPBS under sterile conditions, loose connective tissue was removed and calvaria were washed multiple times. Three calvariae comprised a single biological replicate. Calvariae were digested in collagenase II solution (Gibco; 2mg/ml or 125U/ml) in minimum essential medium **α** (MEM**α)** for 20 min at 37°C, while shaking at 200 rpm. Supernatant was discarded. Another digestion was conducted as above, but this time the supernatant was collected. Calvariae were then washed with 5ml of DPBS, which was also collected, treated with 2ml of 4mM EDTA (Ethylenediaminetetraacetic acid) for 10min, at 37°C, and the supernatant and subsequent DPBS wash collected. The calvariae were further digested with collagenase II solution as before, and the supernatant and the following DPBS wash collected. Supernatant fractions were combined and centrifuged for 5min at 400*g*. Cells were re-suspended in Complete Culture Medium (CCM; MEMα supplemented with 10% filtered FBS, 100U/ml penicillin, 100μg/ml streptomycin, 1.25μg/ml amphotericin B, 2mM L‑glutamine) and Medium was replenished next day. Cells were expanded to ~80‑90% sub-confluency in T‑75 flasks for 3 days following isolation and seeded onto 12- or 24-well plates for differentiation assays.

**Osteoblast differentiation.**

Cells were seeded at 1.25x10^4^ cells/well into 12-well plates (~3.3x10^3^ cells/cm^2^) and allowed to reach up to 100% confluency for an additional 3 days. Osteogenic Medium (CCM supplemented with filter-sterilised 50μg/ml ascorbic acid and 2mM β‑glycerophosphate) was then added to initiate osteogenesis (day 0) (2). Staining assays, sample collection and medium changes were conducted at days 1 and 3, and in some cases, every 3 days thereafter, up to 21 days.

**Osteoblast culture with BCL3 mimetic peptide.**

Calvarial osteoblasts were prepared as above. On the day of Osteogenic Medium addition, 30μM of BCL3 mimetic peptide (BDP2) or mutated peptide (mBDP2) were added to cultures (3). The peptides were synthesised by GenScript: BDP2, YGRKK-RRQRR-AAVYR-ILSLF-KLGSR and mBDP2, YGRKK-RRQRR-WAWGY-ILSLD-CLGSY (3). Medium including peptides was replenished 2 days later and alkaline phosphatase staining assays (described below) were conducted at day 3.

**Quantitative PCR.**

Cells were gently washed thrice with ≥1ml sterile DPBS pre‑warmed to 37°C, then lysed using 700μl of QIAzol Lysis Reagent (QIAGEN). RNA purification was performed as per standard manufacturer instructions using the miRNeasy Mini Kit (QIAGEN). Concentrations of all comparable samples were normalised and cDNA synthesised using the AffinityScript Multiple Temperature cDNA Synthesis Kit (Agilent Technologies), as per manufacturer recommendations. Quantitative PCR (qPCR) was conducted using the Fast SYBR Green Master Mix (Applied Biosystems). Primer pairs used were: *Alpl* (forward, ATTCCCACTATGTCTGGAAC; reverse, CTCAAAGAGACCTAAGAAG), *Actb* (forward, GATGTATGAAGGCTTTGGTC; reverse, TGTGCACTTTTATTGGTCTC), *Col1a1* (forward, GCCAAGAAGACATCCCTGAA; reverse, CTTCCGGGCAGAAAGCA), *Ocn* (forward, ACCATGAGGACCATCTTTC; reverse, GGACATGAAGGCTTTGTC), *Opg* (forward, GAAGATCATCCAAGACATTGAC; reverse, TCCTCCATAAACTGAGTAGC), *Osx* (forward, TGCTTGAGGAAGAAGCTC; reverse, CTTCTTTGTGCCTCCTTTC), *Rankl* (forward, TCTGTTCCTGTACTTTCGAG; reverse, TTCATGGAGTCTCAGGATTC), *Runx2* (forward, GAGAGGTACCAGATGGGACT; reverse, CACTTGGGGAGGATTTGTGA) and *Wnt16* (forward, GACACGAGAGGTGGAACTGTA; reverse, CGCTACTCAGCTCATAGCCAAA).

**RNAseq library preparation and sequencing.**

Using the purified RNA described above, cDNA synthesis was carried out using the SMART-seq v4 Ultra Low Input RNA Kit for Sequencing (Clontech). cDNA was purified using the Agencourt AMPure XP Kit (Beckman Coulter) and fragmented using the Diagenode Bioruptor Pico sonication system. cDNA Fragment size validation was conducted using the Agilent 2100 Bioanalyzer and the High Sensitivity DNA Kit (Agilent). Library preparation was conducted using the Low Input Library Prep Kit HT (Clontech) as per instructions. Library quantification was conducted using the Qubit 3 Fluorometer (ThermoFisher Scientific). Sequencing was conducted using the Illumina HiSeq 4000 System with a read-depth of ~36million/sample, of 75bp paired-end reads. Sequencing, base calling and basic quality control was carried out by Edinburgh Genomics, The University of Edinburgh.

**Bioinformatic analysis.**

Quality control was performed using FastQC (4). Reads were trimmed using Trim Galore!, under default settings and then aligned to the mouse genome (mm10) using TopHat2, under default settings, and per gene read counts were calculated using HTSeq (5-7). Differential expression between the four groups was calculated using DESeq2 (8). Final expression values were represented as fragments per kilobase of exon per million reads (FPKMs) and were calculated using Cufflinks, under default settings (9). Statistical cutoff of adjusted P < 0.05 was considered significant. Downstream analysis utilised the PANTHER gene ontology (GO) enrichment analysis tool (10). Transcription factor binding site (TFBS) analysis was conducted of the region 350bp upstream and 50bp downstream of the gene transcription start site, using HOMER (11). Protein-protein functional association network clustering analysis involved MCL clustering with a 1.2 inflation parameter (12). The transcriptomic data discussed in this study are deposited in the NCBI Gene Expression Omnibus (GEO) and publicly accessible through GEO Series accession number GSE125153 (<https://www.ncbi.nlm.nih.gov/geo/query/acc.cgi?acc=GSE125153>) (13).

**Staining for osteoblast alkaline phosphatase activity.**

Alkaline phosphatase assay was conducted as previously described, using 1‑Step NBT (nitro‑blue tetrazolium chloride) and BCIP (5‑bromo‑4-chloro‑3′-indolyphosphate p‑toluidine salt) substrate solution (ThermoFisher Scientific) (14). Image analysis was conducted via Fiji/ImageJ (version 2.0) packages and involved thresholding of dark pixels (15).

**Sircol staining of osteoblast collagen production.**

With culture medium aspirated, cells were washed thrice with DPBS pre‑warmed to 37°C and fixation was conducted for 20min in 500μl of Bouin’s solution pre‑warmed to 37°C. Fixed cells were washed thrice with dH_2_O and a 500μl volume of Sircol Sirius Red (Biocolor) soluble dye solution was added for 1h to stain collagen. Cells were washed twice with 1ml 0.01M HCl, to remove excess dye and air-dried. Image analysis of staining involved quantification with Fiji/ImageJ (version 2.0).

**Alizarin Red staining of osteoblast mineralisation.**

Cell monolayers were prepared, fixed and mineral nodules stained as previously described (14). Image analysis was conducted via Fiji/ImageJ (version 2.0) packages and involved thresholding of dark pixels (15).

**Osteoclast differentiation and functional assessment.**

Adult female 8-week mice were euthanised by CO_2_ asphyxiation. Hindlimbs were dissected from the hip joint and placed in sterile DPBS. Femurs were dissected, the epiphyseal ends removed with scissors and a bone marrow collected using a needle and syringe into CCM. At day 0, 10ml of suspended bone marrow cells at a density of 1.0x10^6^ cells/ml were cultured overnight with 30ng/ml M‑CSF (R&D Systems). On day 1, non-adherent cells were collected, resuspended at 5.0x10^5^ cells/ml in CCM supplemented with 50ng/ml M-CSF and 50ng/ml RANKL (R&D Systems), and 200μL/well seeded into 96-well plates. Media was replenished at day 5 and on day 6. Tartrate-resistant acid phosphatase (TRAP) staining was performed, using the Acid Phosphatase, Leukocyte (TRAP) Kit (Sigma-Aldrich) according to manufacturer’s instructions. TRAP^+^ cells with ≥3 distinct nuclei were classified as *bona fide* osteoclasts. Area analysis was conducted via Fiji/ImageJ (version 2.0) packages (15). OsteoAssay (Corning) substrate resorption assay was perfomed at day 7.

**Osteoclast culture with BCL3 mimetic peptide.**

Osteoclasts were generated from femoral bone marrow of *Bcl3^−/−^* adult mice aged 14-16 weeks, as described above. 30μM of BCL3 mimetic peptide (BDP2) or mutated BCL3 mimetic peptide (mBDP2) were added on day 1 (3). Cytokine and peptide-supplemented media was replenished at day 5. OsteoAssay (Corning) substrate resorption assay was performed at day 7.

**Osteoblast-osteoclast coculture.**

Adult male 8-week mice were euthanised by CO_2_ asphyxiation. Hindlimbs were dissected from the hip joint and placed in sterile DPBS. Femurs and tibiae were dissected, cleaned of soft tissues and cut into ~1-2mm^3^ pieces. Bone pieces were

washed several times with PBS, and incubated in 4ml collagenase II solution (2mg/ml in MEMα without FBS, filter sterilised) at 37°C in a shaking water bath for 2h, in order to remove all remaining soft tissue and adherent cells. The bone pieces were thoroughly rinsed three times with medium, and transferred to 25 cm^2^ flasks, containing 5ml CCM, at a density of about 20-30 fragments per flask. Media was chaged every three days and after 14 days adherent cultured osteoblasts were isolated and seeded in 96-well plates for co-culture at a density of 25,000 cells/well (~7.8x10^4^ cells/cm^2^). The following day, adult male 8-week mice were euthanised by CO_2_ asphyxiation. Hindlimbs were dissected from the hip joint and placed in sterile DPBS. Femurs and tibiae were dissected and bone marrow suspended using a needle and syringe into CCM. CD14^+^ monocytes were immunomagnetically negatively selected from the bone marrow, using the EasySep Mouse Monocyte Isolation Kit (Stemcell Technologies). Monocytes were co-cultured with osteoblasts at a seeding density of 100,000 cells/well (~3.1x10^5^ cells/cm^2^).

**Micro-CT analysis of adult long bones.**

Whole left deskinned adult legs were fixed in 4% (w/v) formaldehyde solution for ~24 h, followed by immersion and storage in 70% EtOH at 4°C. Samples remained immersed in 70% EtOH during data acquisition in the SkyScan 1272 micro-CT scanner (Bruker). An aluminium 0.5mm energy filter was utilised to acquire non-averaged images with a 0.3° rotation step and a pixel size of 4.5μm. The projection images were processed by a back-projection method using NRecon (version 1.6.10.4). DataViewer (version 1.5.2.4) software enabled alignment of the bone proximodistal axis in the Z-plane of scanned volumes. The ‘tibial trabecular region’ comprised 200 consecutive Z-axis slices, initiating 20 slices distal to the proximal tibial epiphyseal plate, extending distally. The ‘femoral trabecular region’ comprised 200 consecutive slices, initiating 20 slices proximal to the primary spongiosa of the distal epiphyseal plate, extending proximally. The ‘femoral cortical region’ comprised 200 consecutive slices, initiating 420 slices proximal to the primary spongiosa of the distal epiphyseal plate, extending proximally. Specialised macros for the trabecular and cortical regions in the CT Analyser (version 1.16.4.1) software allowed performing of quantitative analysis on the various volumes.

**Biomechanical analyses.**

Right tibiae frozen in H_2_O were defrosted and fibulae and soft tissue removed. Biomechanical 3-point flexure (bend) tests of tibiae were conducted using a 50N load cell device affixed to a calibrated 3-point apparatus (Zwick Roell; BT1‑FR0.5TS.D14). Individual wetted tibiae were situated perpendicularly onto the apparatus, comprising two parallel supporting point-beams separated by 6mm. The tibiae were adjusted to ensure that the tibial crest arched up, the tibial plateau overhang and the tibial ridge faced down to stabilise the bone. Using the accompanying software, testXpert II, the impacting point-beam attached to the load cell was advanced at a rate of 10mm/min and data was recorded until fracture. For the femoral neck break test, femora frozen in H_2_O were defrosted and soft tissue was removed. A machine fitted with a 2kN load cell was used to conduct biomechanical tests (Zwick Roell; 72.0). Proximal halves of femora were retained and the distal, metaphyseal end of the femur was inserted into a screw-adjustable cylindrical holder and was gripped, while the proximal epiphyseal head of the femur protruded out. The holder with bone was affixed at 9° to the vertical, oriented vertically in the sagittal plane and valgus in the frontal plane, approximating orientation *in vivo* (16). It was ensured that the flat-ended indenter contacted only the femoral head and not the greater trochanter. Displacement was applied at a rate of 0.1mm/s until fracture occurred at the femoral neck and the data was recorded.

***In vivo* calcein labelling for dynamic histomorphometry and histology.**

Mice were subjected to dual intraperitoneal injections of 15mg/kg calcein dye (Sigma-Aldrich) solution in DPBS on day -5 and -2, prior to harvest. *Ex vivo* femurs were fixed in 4% (w/v) formaldehyde in DPBS overnight, followed by immersion and storage in 70% EtOH at 4°C. The distal anterior femur was oriented facing up in cassettes. Samples were dehydrated in 2h incubations in serial EtOH concentrations followed by three 6h changes of xylene immersion. A plastic infiltrate solution comprising 89% methyl methacrylate (MMA) resin, 10% dibutyl phthalate plasticiser, 1% Perkadox 16 (AkzoNobel) activator and Tinogard TT (BSAF) antioxidant was prepared. Samples were immersed in two changes of a MMA plastic infiltrate solution in vacuum, for 3h and then 72h, at 4°C. Samples were oriented into sealed moulds containing infiltrate solution and baked at 30°C. Sample sections of ~5μm depth were taken with the Leica RM 2265 microtome from multiple depth-regions. Slides were covered with Kisol foil (Kettenbach GmBH & Co), dried, placed in a section mounting press and baked at 37°C for 2 days. For dynamic histomorphometry, slides were counter-stained for bone with a 1% Calcein Blue (Sigma-Aldrich) for 3min, washed in dH_2_O twice, dehydrated in serial EtOH solutions for 3min each, cleared in xylene for 3min, and finally covered with a glass coverslip with Eukitt mounting medium (Sigma-Aldrich). For histological visualisation, slides of joints were dewaxed in three 20-minute washes of 2-methoxyethyl acetate, cleared in 2 10-minute washes of xylene, rehydrated in decreasing serial dilutions of EtOH, stained for 10 min in a 0.1% solution of Safranin O followed by Fast Green stain, dehydrated in increasing serial dilutions of EtOH and mounted as above. Alternatively, for cathepsin K immunostain, slides were serially hydrated, probed with cathepsin K antibody (Proteintech, 11239-1-AP) and developed with ImmPACT DAB and ImmPRESS Staining kits (Vector Laboratories), according to the manufacturer’s instructions. Slides were visualised using florescent or light microscopy. Intra-label thickness for mineral apposition rate and growth plate height were determined manually using Fiji/ImageJ (version 2.0).

**ELISAs.**

For serum, blood was acquired via terminal cardiac puncture, placed into procoagulant serum separation tubes (BD) and centrifuged at 6,000*g* for 2min to separate serum. P1NP and CTX1 were assessed using RatLaps enzyme immunoassays (Immunodiagnostic Systems); and OPG (R&D Systems), RANKL (R&D Systems) and WNT16 (2B Scieintific Ltd) were also assessed, all according to manufacturer’s instructions.

**Experimental osteoarthritis model.**

Male 10-week mice were aseptically operated under anaesthetic. As part of the destabilisation of the medial meniscus (DMM) procedure, mice received on the left knee (ipsilateral) a ~5mm longitudinal dermal incision lateral to the patella to open the joint capsule (17). The medial meniscotibial ligament (MMTL) was located by conducting a medial para-patellar incision and conducting a blunt dissection of the fat pad over the cranial horn of the medial meniscus (17). Visualisation and transection of the MMTL, was performed, allowing medial displacement of the meniscus for designated mice (17). Mice in the ‘Sham’ groups did not receive an MMTL transection. The joint capsule was sealed with a suture and the cutaneous incision sealed with metal clips. Anaesthetic was discontinued and ~0.10mg/kg buprenorphine was intraperitoneally administered. Mice were monitored daily. Clips were removed 4 days post-procedure and mice analysed 2 weeks after surgery. Micro-CT analysis entailed calculation of bone volume of tibial osteophytes, and volumetric bone density of cross-sectional regions in the medial and lateral tibial subchondral bone plateau and trabecular regions of ipsilateral (left) and contralateral (right) knees (17). Subsequent embedding, sectioning and Safranin O and Fast Green histological analysis (described above) allowed scoring of joint pathology according to Osteoarthritis Research Society International (OARSI)-established guidelines (18).

**Statistical approach.**

Statistical analysis used both the GraphPad Prism (versions 7-9) software and R packages. All data was initially assessed for compliance with a Gaussian distribution, by conducting the D’Augustino-Pearson omnibus normality test. All tests were two-tailed. In data where the sample size (n) was insufficient for a normality test, normality was assumed. In two-group data where the n was insufficient for a normality test, the Student’s *t*‑test with the assumption of an identical standard deviation (SD) was used. For normally-distributed data where two groups were extant, the unpaired *t*‑test with Welch’s correction or the paired ratio *t*‑test was applied. For two-group data failing the normality test, the unpaired Mann-Whitney test was used. Parametric correlations were fitted with linear regression, while non-parametric correlations were subject to Spearman’s r correlation test. The extra sum-of-squares F test was specifically used to test differences between two nested sets of curves. Data with multiple groups and a single variable were compared using analysis of variance (ANOVA), followed by multiple comparisions using Fisher’s least significant difference test. *P*-values less than 0.05 were considered significant, throughout. Complex dataset analyses were performed by the specific tools and packages described elsewhere, subject to the statistical tests described in their published literature, and multiplicity-adjusted *P*-values were rendered.

**References for Supplementary Online Materials and Methods.**

1. Rigueur D, Lyons KM. Whole-mount skeletal staining. *Methods Mol Biol* 2014;1130:113–121.

2. Orriss IR, Hajjawi MOR, Huesa C, MacRae VE, Arnett TR. Optimisation of the differing conditions required for bone formation in vitro by primary osteoblasts from mice and rats. *Int J Mol Med* 2014;34:1201–1208.

3. Collins PE, Grassia G, Colleran A, Kiely PA, Ialenti A, Maffia P, et al. Mapping the Interaction of B Cell Leukemia 3 (BCL-3) and Nuclear Factor κB (NF-κB) p50 Identifies a BCL-3-mimetic Anti-inflammatory Peptide. *J Biol Chem* 2015;290:15687–15696.

4. Leggett RM, Ramirez-Gonzalez RH, Clavijo BJ, Waite D, Davey RP. Sequencing quality assessment tools to enable data-driven informatics for high throughput genomics. *Front Genet* 2013;4:288.

5. Lindgreen S. AdapterRemoval: easy cleaning of next-generation sequencing reads. *BMC Res Notes* 2012;5:337.

6. Kim D, Pertea G, Trapnell C, Pimentel H, Kelley R, Salzberg SL. TopHat2: accurate alignment of transcriptomes in the presence of insertions, deletions and gene fusions. *Genome Biol* 2013;14:R36.

7. Anders S, Pyl PT, Huber W. HTSeq—a Python framework to work with high-throughput sequencing data. *Bioinformatics* 2015;31:166–169.

8. Love MI, Huber W, Anders S. Moderated estimation of fold change and dispersion for RNA-seq data with DESeq2. *Genome Biol* 2014;15:550.

9. Trapnell C, Roberts A, Goff L, Pertea G, Kim D, Kelley DR, et al. Differential gene and transcript expression analysis of RNA-seq experiments with TopHat and Cufflinks. *Nat Protoc* 2012;7:562–578.

10. Mi H, Huang X, Muruganujan A, Tang H, Mills C, Kang D, et al. PANTHER version 11: expanded annotation data from Gene Ontology and Reactome pathways, and data analysis tool enhancements. *Nucleic Acids Res* 2017;45:D183–D189.

11. Heinz S, Benner C, Spann N, Bertolino E, Lin YC, Laslo P, et al. Simple combinations of lineage-determining transcription factors prime cis-regulatory elements required for macrophage and B cell identities. *Mol Cell* 2010;38:576–589.

12. Szklarczyk D, Gable AL, Lyon D, Junge A, Wyder S, Huerta-Cepas J, et al. STRING v11: protein–protein association networks with increased coverage, supporting functional discovery in genome-wide experimental datasets. *Nucleic Acids Res* 2018;47:D607–D613.

13. Barrett T, Wilhite SE, Ledoux P, Evangelista C, Kim IF, Tomashevsky M, et al. NCBI GEO: archive for functional genomics data sets--update. *Nucleic Acids Res* 2013;41:D991–5.

14. Jonason JH, O’Keefe RJ. Isolation and Culture of Neonatal Mouse Calvarial Osteoblasts. In: *Skeletal Development and Repair*.Vol 1130. Methods in Molecular Biology. Totowa, NJ: Humana Press, Totowa, NJ; 2014:295–305.

15. van 't Hof RJ, Rose L, Bassonga E, Daroszewska A. Open source software for semi-automated histomorphometry of bone resorption and formation parameters. *Bone* 2017;99:69–79.

16. Hessle L, Stordalen GA, Wenglén C, Petzold C, Tanner EK, Brorson S-H, et al. The skeletal phenotype of chondroadherin deficient mice. *PLoS ONE* 2013;8:e63080.

17. Huesa C, Ortiz AC, Dunning L, McGavin L, Bennett L, McIntosh K, et al. Proteinase-activated receptor 2 modulates OA-related pain, cartilage and bone pathology. *Ann Rheum Dis* 2016;75:1989–1997.

18. Glasson SS, Chambers MG, Van Den Berg WB, Little CB. The OARSI histopathology initiative - recommendations for histological assessments of osteoarthritis in the mouse. *Osteoarthr Cartil*. 2010 Oct;18 Suppl 3:S17-23.
