# Supplementary figures and images for: IĸB Protein BCL3 as a Controller of Osteogenesis and Bone Health
Source: Arthritis Rheumatol. 2023 Oct 1;75(12):2148–60. doi: 10.1002/art.42639 (PMC10952620; doi:10.1002/art.42639)

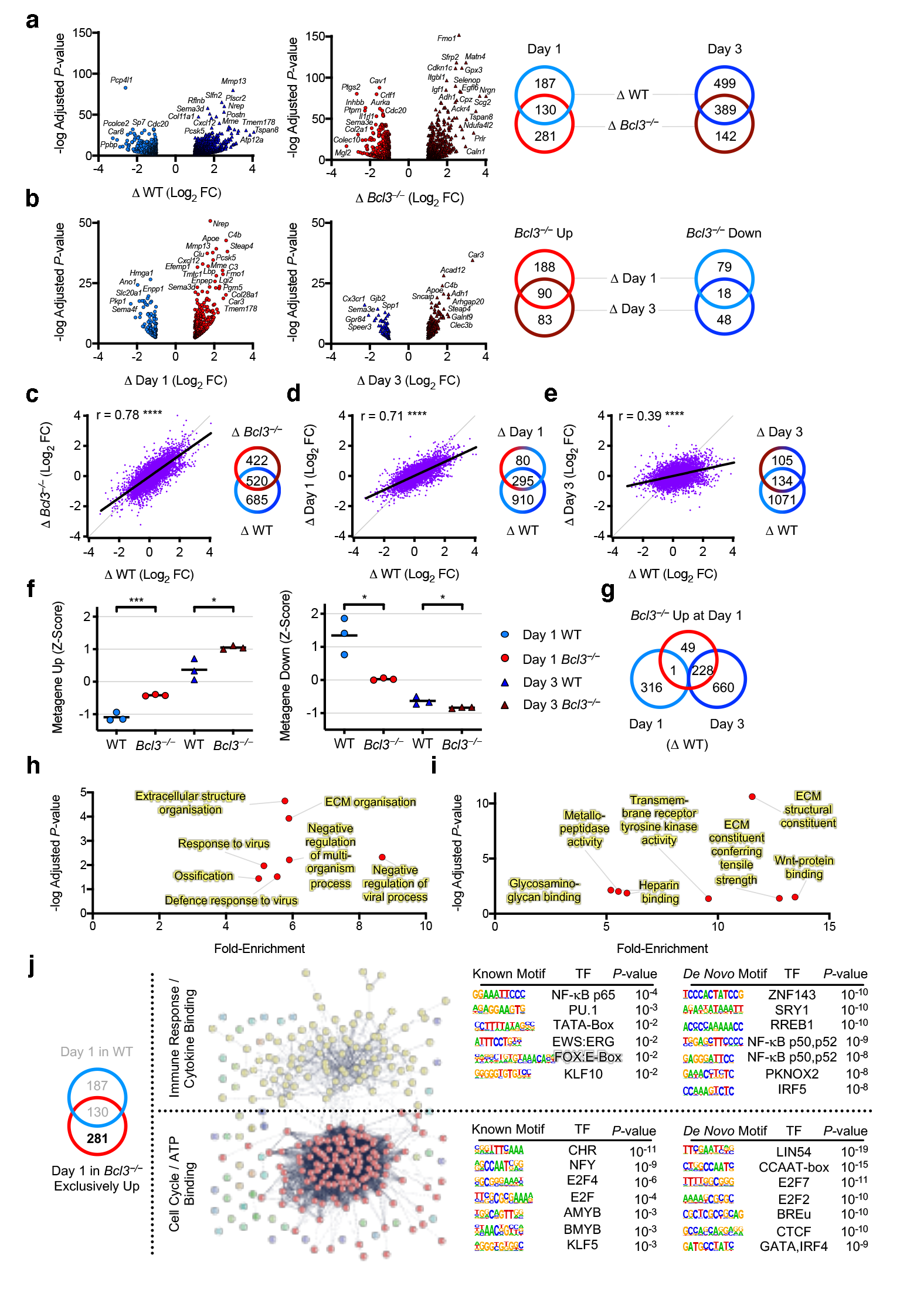

Supplement: Supplementary file 4 — Supplementary Figure 1: RNA‐seq transcriptomic profiling of WT and Bcl3 −/− osteoblasts at day 1 and day 3 following osteogenic induction. a, Significantly (P adj < 0.05) differentially expressed genes between day 1 and day 3, with > 1 absolute fold‐change (FC) in both WT and Bcl3 −/− cells. Venn diagrams illustrate shared or differentially expressed genes in both genotypes at day 1 and day 3. b, Significantly (P adj < 0.05) differentially expressed genes in Bcl3 −/− cells, relative to WT, with > 1 absolute fold‐change (FC) at day 1 and day 3. Venn diagrams illustrate sustained or transient upregulated and downregulated genes in Bcl3 −/− cells. c‐e, Correlations of gene expression fold‐changes (FC) through time, from day 1 to day 3, in WT (Δ WT) and Bcl3 −/− (Δ Bcl3 −/− ) genotypes during osteogenesis (c), between Δ WT and Bcl3 −/− genes at day 1 (Δ Day 1) compared to WT controls (d), and between Δ WT and Bcl3 −/− genes at day 3 (Δ Day 3) compared to WT controls (e). Venn diagrams to the right of each panel indicate significantly different genes (P adj < 0.05, fold‐change ± 1) to corresponding datasets on each axis. Spearman's rank correlation coefficient, r. n = 14493. f, Median expression level Z‐scores of upregulated (left) and downregulated (right) gene aggregates (metagene) in experimental replicates, relative to the gene expression change in WT cells between days 1 and 3 (Δ WT). n = 3. Student's t‐test for pairwise comparisons. g, Genes upregulated in Bcl3 −/− cells at day 1 (light red circle), compared to genes differentially expressed between day 1 (light blue circle) and day 3 (dark blue circle) in WT cells. Overlapping regions represent shared gene‐sets. h‐i, Gene ontology (GO) analysis of biological process (h) and molecular function (i) of genes upregulated in Bcl3 −/− cells at day 1, compared to WT. The top seven results in order of fold enrichment are highlighted. j, Genes significantly upregulated at day 1, exclusively in Bcl3 −/− cells (‘281’ transcr [file ART-75-2148-s009.tif]

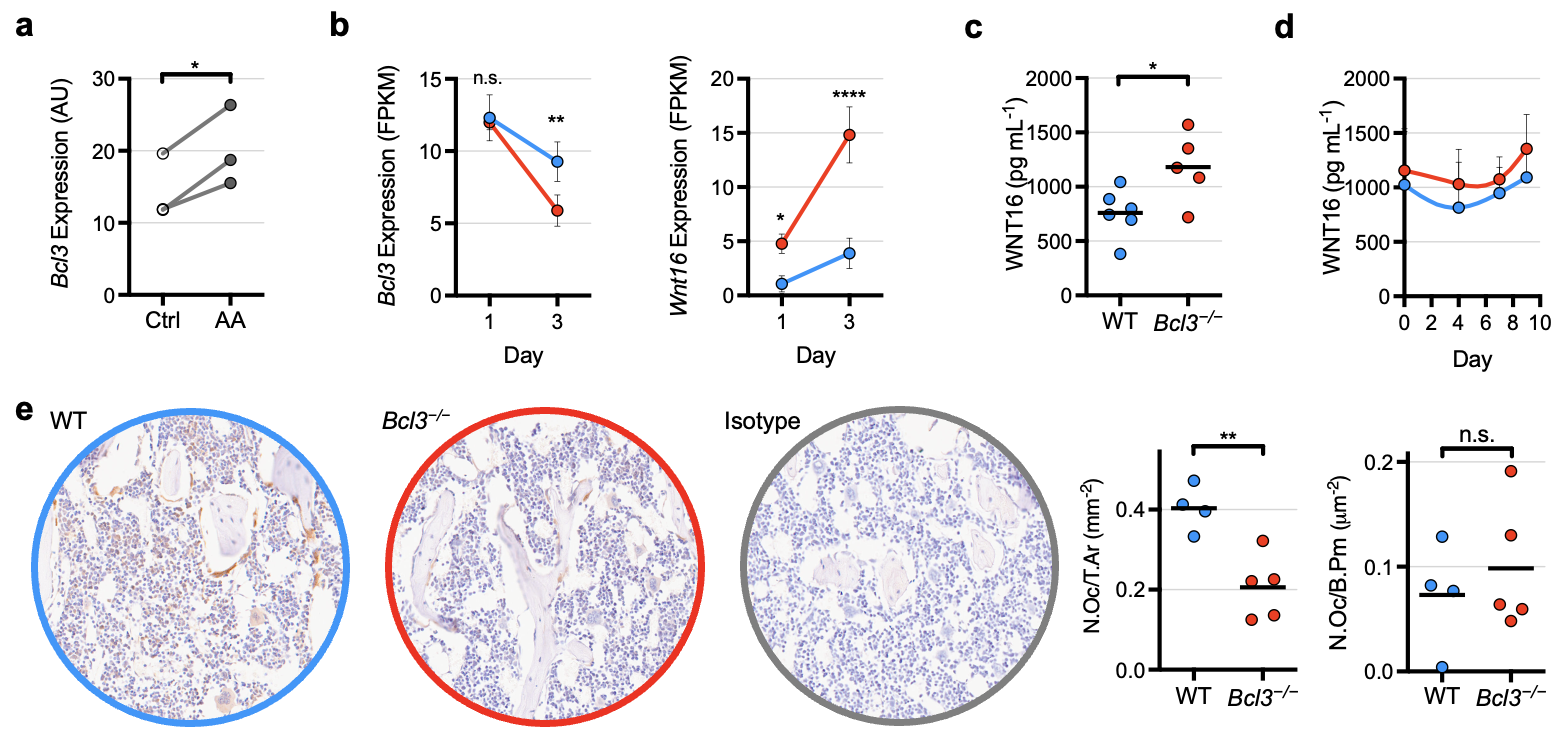

Supplement: Supplementary file 5 — Supplementary Figure 2: Characterisation of Bcl3 and Wnt16 expression in osteoblasts, WNT16 protein in osteoblast‐osteoclast co‐cultures and osteoclast activity in adult bone of WT and Bcl3 −/− mice. a, Bcl3 gene expression in control and ascorbic acid (AA) stimulated murine MC3T3‐E1 osteoblast cells at day 5 (GEO accession: GSE37676). n = 3. b, Bcl3 and Wnt16 transcript expression (RNA‐seq mapped) in WT and Bcl3 −/− osteoblasts at days 1 and 3. n = 3. c, WNT16 protein levels in osteoblast culture supernatants at day 9, without monocytes/osteoclasts. n = 5‐6. d, WNT16 levels in osteoblast‐osteoclast co‐culture supernatants until day 9. n = 5‐6. e, Representative images and quantification of cathepsin K (CTSK) stained sections, in brown, of tibiae from adult 12‐week WT and Bcl3 −/− mice. Image diameter = 400μm. Number of osteoclasts (N.Oc) per tissue area (T.Ar) and number of osteoclasts per bone perimeter (B.Pm). n = 4‐5. Student's t‐test (a, c and e) or Two‐way ANOVA (b and d). * P < 0.05, ** P < 0.01, **** P < 0.0001 and ‘n.s.’ ‐ not significant. [file ART-75-2148-s010.tiff]

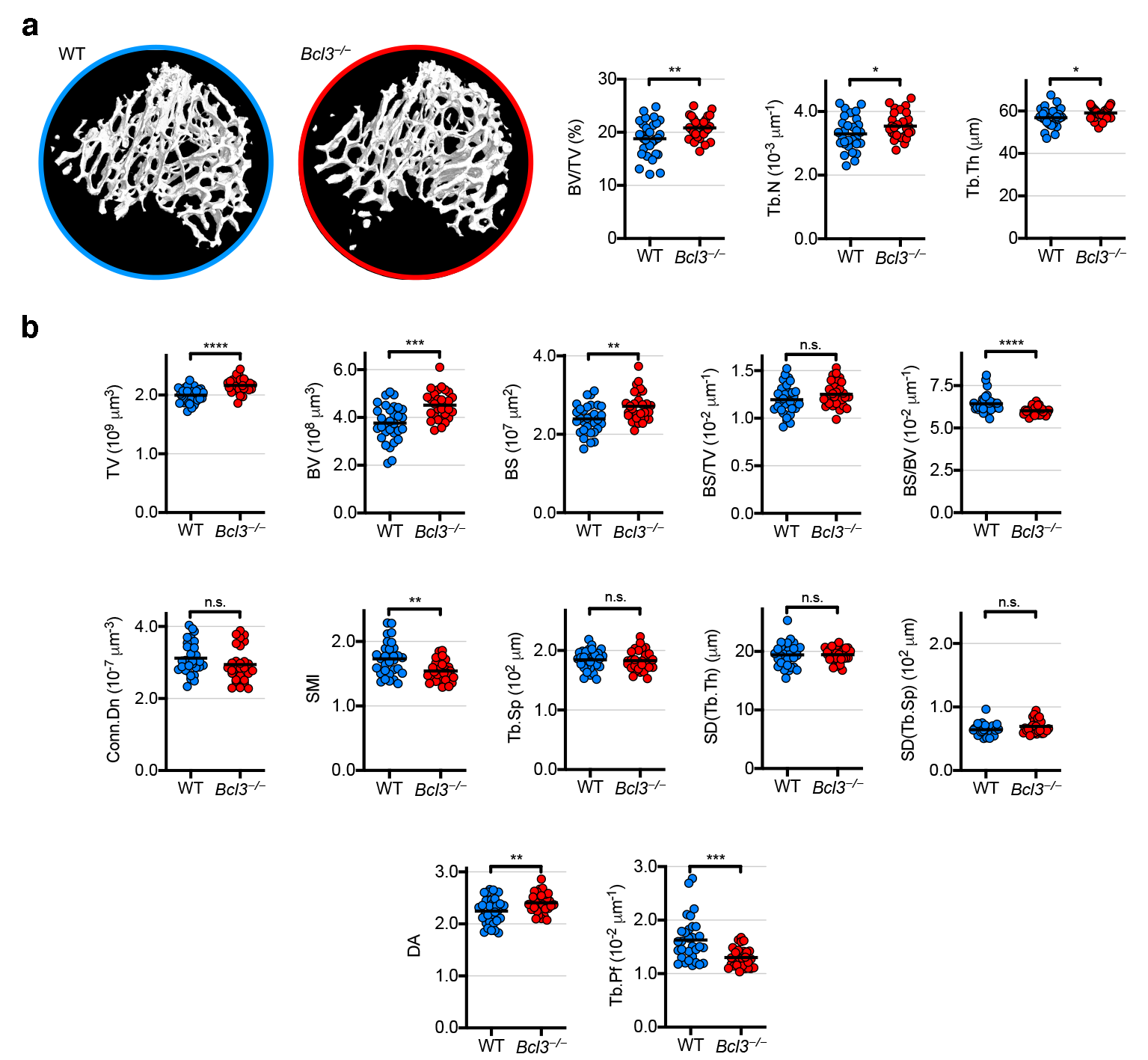

Supplement: Supplementary file 6 — Supplementary Figure 3: Additional parameters of the femoral trabecular region of 20‐week WT and Bcl3 −/− male mice, including tissue volume (TV), bone volume (BV), bone surface (BS), bone surface density (BS/TV), bone surface/volume ratio (BS/BV), connectivity density (Conn.Dn), structural model index (SMI), trabecular separation (Tb.Sp), standard deviation of trabecular thickness [SD(Tb.Th)], standard deviation of trabecular separation [SD(Tb.Sp)], degree of anisotropy (DA) trabecular pattern factor (Tb.Pf). n = 29‐31. Welch's t‐test or Mann‐Whitney test. ** P < 0.01, *** P < 0.001, **** P < 0.0001 and ‘n.s.’ ‐ not significant. [file ART-75-2148-s007.zip › Suppl_Fig_3_nl.tif]

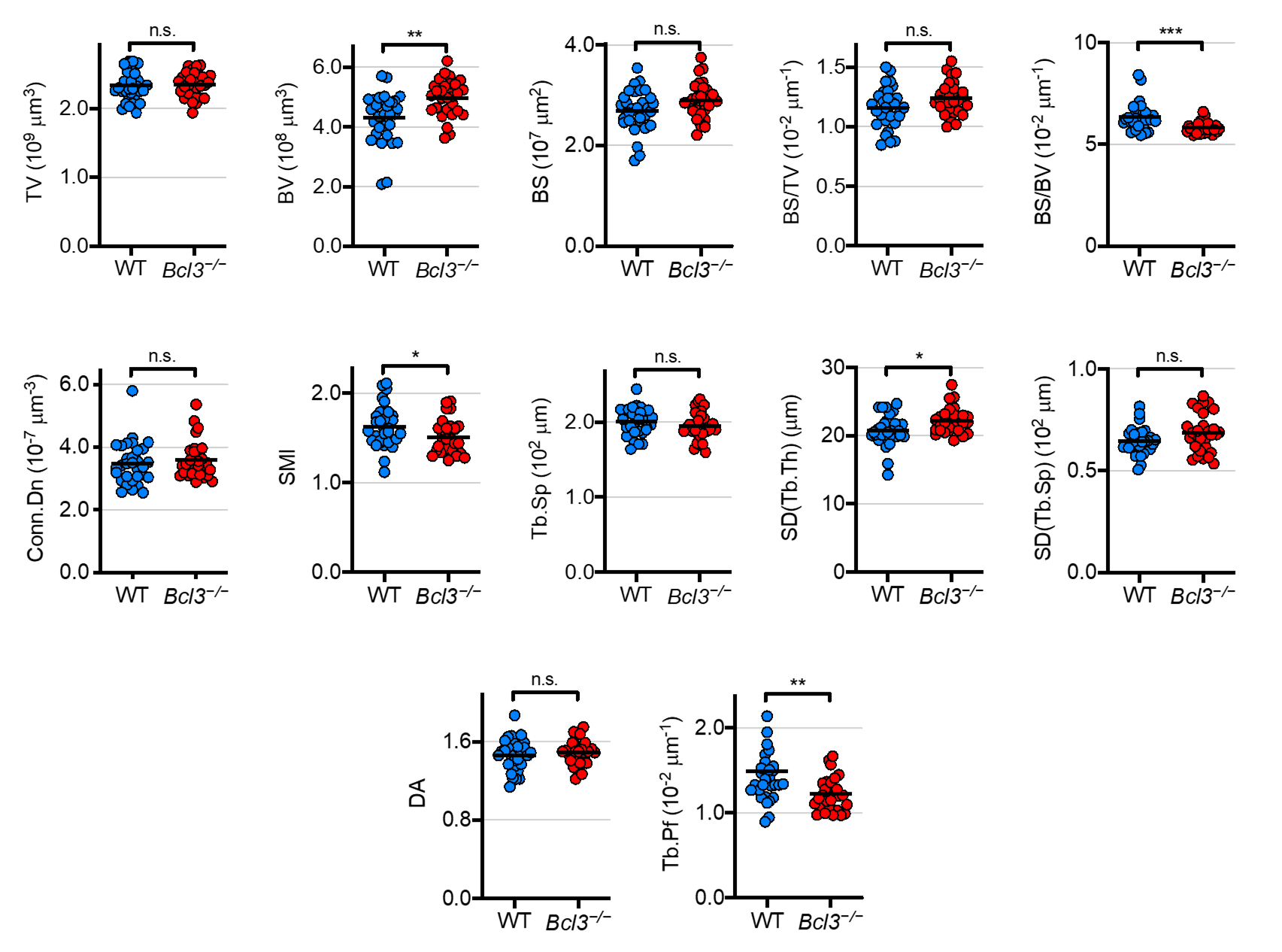

Supplement: Supplementary file 6 — Supplementary Figure 3: Additional parameters of the femoral trabecular region of 20‐week WT and Bcl3 −/− male mice, including tissue volume (TV), bone volume (BV), bone surface (BS), bone surface density (BS/TV), bone surface/volume ratio (BS/BV), connectivity density (Conn.Dn), structural model index (SMI), trabecular separation (Tb.Sp), standard deviation of trabecular thickness [SD(Tb.Th)], standard deviation of trabecular separation [SD(Tb.Sp)], degree of anisotropy (DA) trabecular pattern factor (Tb.Pf). n = 29‐31. Welch's t‐test or Mann‐Whitney test. ** P < 0.01, *** P < 0.001, **** P < 0.0001 and ‘n.s.’ ‐ not significant. [file ART-75-2148-s007.zip › Suppl_figure_3.TIF]

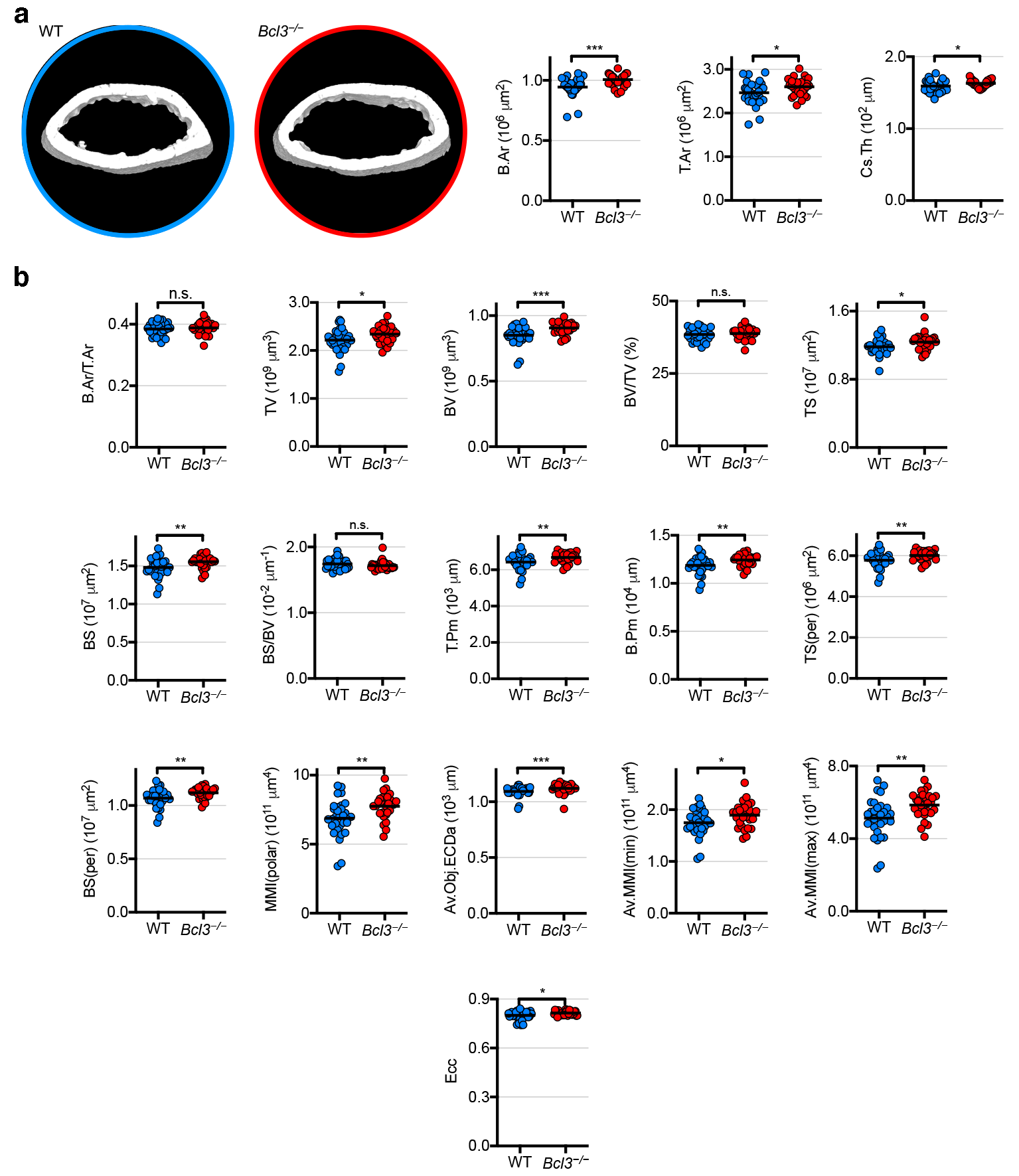

Supplement: Supplementary file 7 — Supplementary Figure 4: Phenotypic analyses of 20‐week WT and Bcl3 −/− male mice proximal tibial trabecular region. a, Representative volumetric bone density visualisations (images not to scale) and key three‐dimensional morphometric parameters, including percent bone volume density (BV/TV), trabecular number (Tb.N), and trabecular thickness (Tb.Th). b, Additional parameters of the tibial trabecular region of 20‐week WT and Bcl3 −/− male mice, including tissue volume (TV), bone volume (BV), bone surface (BS), bone surface density (BS/TV), bone surface/volume ratio (BS/BV), connectivity density (Conn.Dn), structural model index (SMI), trabecular separation (Tb.Sp), standard deviation of trabecular thickness [SD(Tb.Th)], standard deviation of trabecular separation [SD(Tb.Sp)], degree of anisotropy (DA) trabecular pattern factor (Tb.Pf). n = 29‐31. Welch's t‐test or Mann‐Whitney test. * P < 0.05, ** P < 0.01, *** P < 0.001, **** P < 0.0001 and ‘n.s.’ ‐ not significant. [file ART-75-2148-s003.tif]

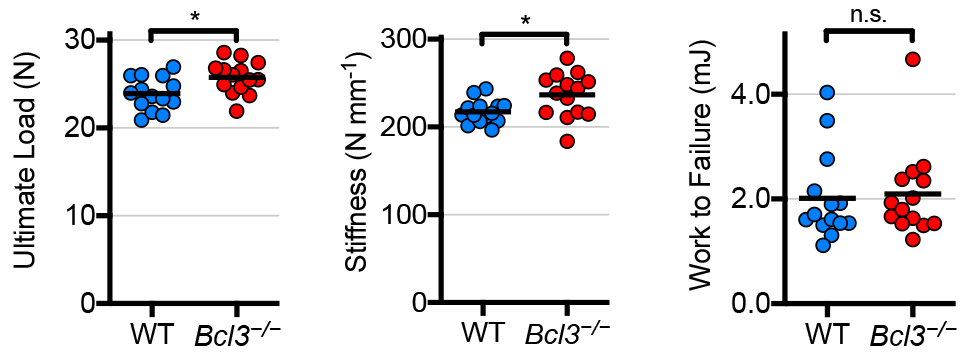

Supplement: Supplementary file 8 — Supplementary Figure 5: Phenotypic analyses of 20‐week WT and Bcl3 −/− male mice distal femoral cortical region. a, Representative volumetric bone density visualisations (images not to scale) and key two‐dimensional parameters, including mean total cross‐sectional bone area (B.Ar), mean total cross‐sectional tissue area (T.Ar) and cross‐sectional thickness (Cs.Th). b, Additional parameters of the femoral cortical region of 20‐week WT and Bcl3 −/− male mice, including cortical area fraction (B.Ar/T.Ar), tissue volume (TV), bone volume (BV), percent bone volume (BV/TV), tissue surface (TS), bone surface (BS), bone surface/volume ratio (BS/BV), mean total cross‐sectional tissue perimeter (T.Pm), mean total cross‐sectional bone perimeter (B.Pm), peripheral tissue surface [TS(per)], peripheral bone surface [BS(per)], mean polar moment of inertia [MMI(polar)], average object equivalent circle diameter per slice (Av.Obj.ECDa), average principal moment of inertia minimum [Av.MMI(min)], average principal moment of inertia maximum [Av.MMI(max)] and eccentricity (Ecc). n = 29‐31. Welch's t‐test or Mann‐Whitney test. * P < 0.05, ** P < 0.01, *** P < 0.001 and ‘n.s.’ ‐ not significant. [file ART-75-2148-s004.tiff]

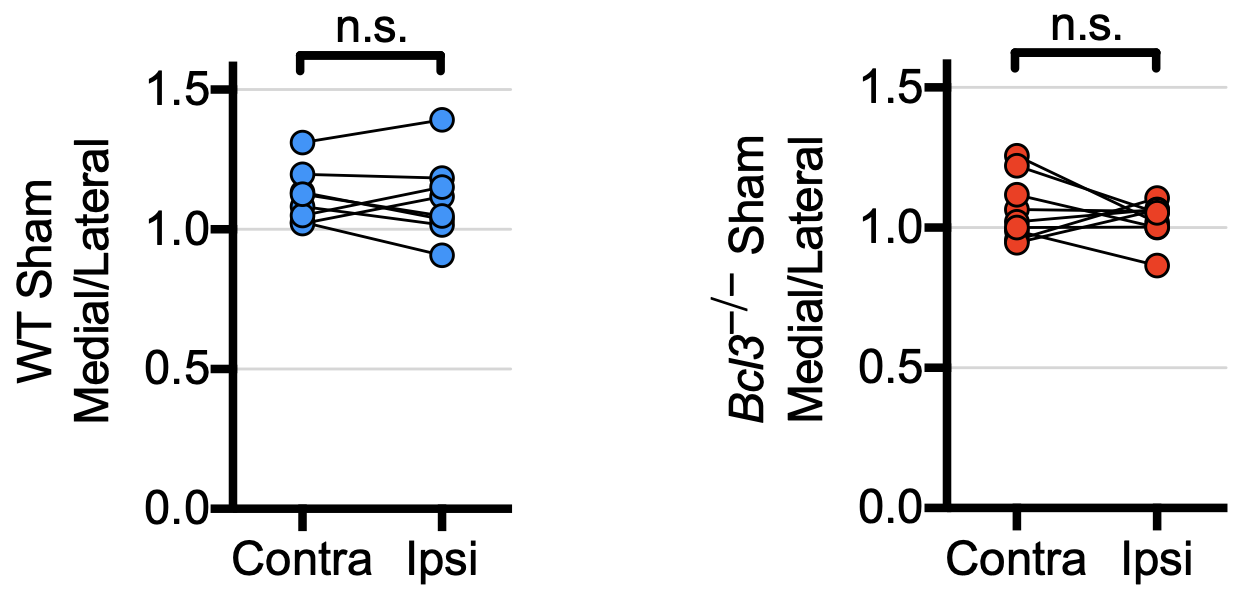

Supplement: Supplementary file 9 — Supplementary Figure 6: Biomechanical outputs of the femoral neck break test of 20‐week WT and Bcl3 −/− male mice. n = 14. Welch's t‐test or Mann‐Whitney test. * P < 0.05 and ‘n.s.’ ‐ not significant. [file ART-75-2148-s002.tiff]
